# Renal and vascular effects of kallikrein inhibition in a model of *Lonomia obliqua* venom-induced acute kidney injury

**DOI:** 10.1371/journal.pntd.0007197

**Published:** 2019-02-14

**Authors:** Markus Berger, João Alfredo de Moraes, Walter Orlando Beys-da-Silva, Lucélia Santi, Paula Barros Terraciano, David Driemeier, Elizabeth Obino Cirne-Lima, Eduardo Pandolfi Passos, Maria Aparecida Ribeiro Vieira, Thereza Christina Barja-Fidalgo, Jorge Almeida Guimarães

**Affiliations:** 1 Laboratório de Bioquímica Farmacológica, Centro de Pesquisa Experimental (CPE), Hospital de Clínicas de Porto Alegre (HCPA-UFRGS), Porto Alegre, RS, Brazil; 2 Programa de Pós-Graduação em Ciências de Saúde: Ginecologia e Obstetrícia (PPGGO), Faculdade de Medicina, Universidade Federal do Rio Grande do Sul (UFRGS), Porto Alegre, RS, Brazil; 3 Laboratório de Biologia REDOX, Instituto de Ciências Biomédicas, Universidade Federal do Rio de Janeiro (UFRJ), Rio de Janeiro, RJ, Brazil; 4 Laboratory of Cellular and Molecular Pharmacology, IBRAG, Universidade do Estado do Rio de Janeiro (UERJ), Rio de Janeiro, RJ, Brazil; 5 Faculdade de Farmácia, Universidade Federal do Rio Grande do Sul (UFRGS), Porto Alegre, RS, Brazil; 6 Laboratório de Embriologia e Diferenciação Celular, Centro de Pesquisa Experimental (CPE), Hospital de Clínicas de Porto Alegre (HCPA-UFRGS), Porto Alegre, RS, Brazil; 7 Departamento de Patologia Clínica Veterinária, Faculdade de Medicina Veterinária, Universidade Federal do Rio Grande do Sul (UFRGS), Porto Alegre, RS, Brazil; 8 Laboratório de Fisiologia Renal, Departamento de Fisiologia e Biofísica, Instituto de Ciências Biológicas (ICB), Universidade Federal de Minas Gerais (UFMG), Belo Horizonte, MG, Brazil; 9 Programa de Pós-Graduação em Biologia Celular de Molecular (PPGBCM), Centro de Biotecnologia (Cbiot-UFRGS), Universidade Federal do Rio Grande do Sul (UFRGS), Porto Alegre, RS, Brazil; Instituto Butantan, BRAZIL

## Abstract

**Background:**

*Lonomia obliqua* venom is nephrotoxic and acute kidney injury (AKI) is the main cause of death among envenomed victims. Mechanism underlying *L*. *obliqua*-induced AKI involves renal hypoperfusion, inflammation, tubular necrosis and loss of glomerular filtration and tubular reabsorption capacities. In the present study, we aimed to investigate the contribution of kallikrein to the hemodynamic instability, inflammation and consequent renal and vascular impairment

**Methodology/Principal findings:**

Addition of *L*. *obliqua* venom to purified prekallikrein and human plasma *in vitro* or to vascular smooth muscle cells (VSMC) in culture, was able to generate kallikrein in a dose-dependent manner. Injected in rats, the venom induced AKI and increased kallikrein levels in plasma and kidney. Kallikrein inhibition by aprotinin prevented glomerular injury and the decrease in glomerular filtration rate, restoring fluid and electrolyte homeostasis. The mechanism underlying these effects was associated to lowering renal inflammation, with decrease in pro-inflammatory cytokines and matrix metalloproteinase expression, reduced tubular degeneration, and protection against oxidative stress. Supporting the key role of kallikrein, we demonstrated that aprotinin inhibited effects directly associated with vascular injury, such as the generation of intracellular reactive oxygen species (ROS) and migration of VSMC induced by *L*. *obliqua* venom or by diluted plasma obtained from envenomed rats. In addition, kallikrein inhibition also ameliorated venom-induced blood incoagulability and decreased kidney tissue factor expression

**Conclusions/Significance:**

These data indicated that kallikrein and consequently kinin release have a key role in kidney injury and vascular remodeling. Thus, blocking kallikrein may be a therapeutic alternative to control the progression of venom-induced AKI and vascular disturbances.

## Author summary

The envenomation by venomous animals is considered a common and serious occupational disease, especially in rural areas of tropical developing countries. Its public health relevance has been largely ignored by medical authorities worldwide. Specifically in regions of southern and southeast Brazil, accidents with the venomous caterpillar *Lonomia obliqua* have been an emergent problem, not only for their high incidence rates, but also by the severity of the clinical consequences observed in envenomation cases. Frequently, envenomed victims present a severe hemorrhagic syndrome that can progress to acute kidney injury (AKI) and death. *L*. *obliqua* venom is highly nephrotoxic, AKI is the main cause of death between patients and its mechanisms are poorly understood. Even after the introduction of antivenom therapy in 1995, there was no reduction in the incidence of AKI. In this work we investigated the contribution of kallikrein to the hemodynamic instability, inflammation and consequent renal and vascular impairment during envenomation. A multidisciplinary approach was employed including methods of renal physiology/biochemistry, morphological and cell culture analysis. We believe the data presented here give new insights to the actual knowledge presenting kallikrein and the kinin system as key points to control the progression of renal disease, which may be explored as an approach complementary to serotherapy.

## Introduction

Envenomation resulting from contact with *Lonomia obliqua* caterpillars has been recognized as a neglected public health issue that mainly occurs in impoverished communities in the rural areas of the southern regions of Brazil. Envenomed victims display systemic hemorrhage secondary to intravascular disseminated coagulation **[**for review see **1,2]**. Frequently, the clinical profile evolves to acute kidney injury (AKI), which is the main cause of death following this type of envenomation **[3–5]**. In experimental models, *L*. *obliqua* venom induces the sudden loss of basic renal functions, including filtration and excretion capacities, urinary concentration and the maintenance of body fluid homeostasis. In addition to the direct cytotoxic effects of venom toxins, renal hypoperfusion also appears to be an important underlying mechanism, because signs of glomerular fibrin deposition and hemodynamic instability (systemic hypotension and increased renal vascular permeability) have been detected in rats injected with *L*. *obliqua* venom **[6]**. In fact, the coagulation, complement and kallikrein-kinin systems are known to be activated during envenomation **[7,8]**.

The kallikrein-kinin system (KKS) is composed of proteolytic enzymes and their substrates, being able to generate potent vasoactive and proinflammatory molecules that are involved in the control of blood pressure, vascular permeability, vascular smooth muscle cell contraction or relaxation, and pain. Both tissue and plasma kallikreins are key elements in this system, since they generated kinins by the proteolytic cleavage of kininogens. At the same time, kallikrein can also directly convert FXII to its active form (FXIIa) leading to auto activation of the kinin system and thrombus formation via the intrinsic pathway **[9]**. Once released, kinins exert the majority of their biological effects by activating two types of kinin receptors: the bradykinin B_1_ receptor (B_1_R) and the bradykinin B_2_ receptor (B_2_R). The B_2_R is constitutively expressed in most tissues and displays a higher affinity for bradykinin (BK) and Lys-BK peptides. In contrast, the B_1_R displays high affinities for the kinin metabolites des-Arg^9^-BK and Lys-des-Arg^9^-BK. The B_1_R is not expressed under normal conditions, but is induced following inflammatory, infectious or traumatic stimuli **[10–13]**. Notably, kallikrein and kinin receptors (B_1_R and B_2_R) are involved in several inflammation-related processes, such as atherosclerosis, airway inflammation, diabetic neuropathy, inflammatory bowel disease, neuropathic pain, cerebral infarction and stroke **[14–20]**. Blocking of distinct members of KKS reduced vascular leakage, inflammation and thrombosis in different experimental models **[21]**.

The KKS is involved in the edematogenic and hypotensive responses elicited by *L*. *obliqua* venom. The cattrepillar’s venom induces the release of kinins from low-molecular weight kininogen (LMWK), and both edematogenic and hypotensive responses are reduced following kallikrein inhibition and B_2_R antagonism **[8]**. Envenomed patients presented low levels of plasma prekallikrein, indicating that kallikrein had been activated and released into the blood circulation **[7]**. Experimental data have also shown that venom components can be localized in vascular smooth muscle cells (VSMC) *in vivo*, and *in vitro*, *L*. *obliqua* venom can trigger reactive oxygen species (ROS) via NADPH oxidase activation **[22,6]**. Taken together, the data led us to hypothesize that the generation of kallikrein during envenomation may be involved in *L*. *obliqua*-induced hemodynamic instability, inflammation, ROS production and the consequent renal functional and vascular impairment.

In the present study, we used a pharmacological approach to study the roles of kallikrein in an experimental model of *L*. *obliqua*-induced AKI. The results indicated that kallikrein inhibition prevented venom-induced blood incoagulability, vascular injury and restore renal function by reducing tubular necrosis, tissue inflammation and ROS generation, which was confirmed through *in vitro* analysis in VSMC. In summary, data indicate that pharmacological blockage of kallikrein activity could be a therapeutic alternative to be used to control the progression of the critical renal pathology installed following *L*. *obliqua* envenomation.

## Materials and methods

### Drugs and reagents

Vanadium (III) chloride (VCl_3_), sulfanilamide (SULF), *N*-(1-naphthyl) ethylenediamine dihydrochloride (NEDD), 3-(4,5-Dimethylthiazol-2-yl)-2,5-diphenyltetrazolium bromide (MTT), purified coagulation factors (VII, IX, X, fibrinogen, plasminogen and kallikrein), Soya Bean Trypsin Inhibitor (SBTI), Dulbecco’s modified Eagle’s medium (DMEM) and fetal calf serum (FCS) were purchased from Sigma-Aldrich (Saint Louis, MO, USA). Aprotinin (Trasylol^®^) was obtained from Bayer do Brasil S.A. (São Paulo, Brazil). The chromogenic substrates for factor Xa (S2222, Bz-Ile-Glu-Gly-Arg-p-nitroanilide), plasma kallikrein (S2302, H-D-Pro-Phe-Arg-p-nitroanilide) and tissue kallikrein (S2366, H-D-Val-Leu-Arg-p-nitroanilide) were obtained from Chromogenix (Milan, Italy). CM-H2DCFDA (DCF), penicillin/streptomycin, EDTA and trypsin were from Life Technologies (Carlsbad, CA, USA). Ketamine and xylazine were obtained from Syntec (São Paulo, Brazil). Anti-lonomic serum was kindly donated by Butantan Institute (São Paulo, Brazil).

### Venom

*L*. *obliqua* caterpillars were kindly provided by the Centro de Informações Toxicológicas (CIT), Porto Alegre, Rio Grande do Sul, Brazil. The specimens used in the present study were collected in the cities of Bom Princípio and Progresso, Rio Grande do Sul, Brazil. *L*. *obliqua* venom was obtained by cutting bristles at the base of each scoli and macerating them in cold phosphate-buffered saline (PBS), pH = 7.4, as previously described **[23–25]**. The venom obtained following this procedure was designated as ***L****onomia* ***o****bliqua* **B**ristle **E**xtract (LOBE). The protein concentrations of LOBE samples were determined using a BCA assay kit (Pierce, Rockford, USA), and the aliquots were stored at -80°C prior to use.

### In vitro studies

#### Plasma prekallikrein and prothrombin and factor X deficient plasma preparation

Plasma prekallikrein was purified from rat plasma through steps of ammonium sulfate precipitation followed by chromatographic steps on DEAE, heparin and CM-sepharose (for details see supplementary methods, S1 Methods). The homogeneity of this preparation was analyzed by SDS-PAGE 12% under reducing conditions with 2-mercaptoethanol and PPKLK was identified by immunoblot using a rabbit anti-rat polyclonal PKLK antibody (1:2000, Cusabio Biotech Co., Wuhan, China). A human plasma depleted in prothrombin and factor X (plasma -PThr/-FX) was prepared from a commercially available factor X deficient plasma (Sigma-Aldrich, Saint Louis, MO, USA) which was adsorbed three times in barium sulfate to completely remove prothrombin (a detailed description is provided in supplementary methods, S1 Methods).

#### *L*. *obliqua* venom–induced prekallikrein activation

*L*. *obliqua* induced kallikrein generation was measured *in vitro* using purified rat plasma prekallikrein. Briefly, different concentrations of LOBE (1, 3, 10, 50, 100 and 200 μg/mL) were incubated during 30 min in the presence or absence of plasma prekallikrein (25 or 50 μg) in 50 mM Tris-HCl, pH 7.5 containing 150 mM NaCl (final volume of 100 μL), at 37 ^o^C. After that, kallikrein, factor Xa and thrombin–like activities were measured by the addition (0.2 mM, final concentration) of chromogenic synthetic substrates S-2302, S-2222 or S2238, respectively. The kinetics of *p*-nitroaniline formation were monitored (405 nm) at a time interval of 14 s during a total time of 30 min on the microplate reader spectrophotometer (SpectraMAX 190, Molecular Devices, Sunnyvale, CA, USA). Results are expressed as mOD/min and represent the mean ± standard error of three independent experiments.

Alternatively, *L*. *obliqua*–induced prekallikrein activation was also confirmed by SDS-PAGE, immunoblot and zymography. Purified PPKLK (25 μg) was incubated in the presence or absence of LOBE (5 μg) during 15 min or 30 min and KLK formation was then analyzed by SDS-PAGE 12% or western-blot both running under reducing conditions. PKLK/KLK band was specifically identified using a rabbit anti-rat polyclonal PKLK antibody (1:2000, Cusabio Biotech Co., Wuhan, China). The activity of generated KLK was also detected based on its property to activate plasminogen using zymogram gels. In this case, samples were loaded onto non-reducing 12% SDS–polyacrylamide gels containing 1 mg/mL casein and 10 μg/mL plasminogen, as previously described **[26]**.

#### *L*. *obliqua* venom–induced kallikrein formation in human plasma

Normal human plasma was diluted (1:10) in phosphate buffered saline (PBS) and incubated with different concentrations of LOBE (1, 3, 10, 50, 100 and 200 μg/mL) in the presence of 100 nM SBTI in a final volume of 100 μL, at 37 ^o^C. Aliquots of 10 μL were taken and generated kallikrein enzymatic activity was determined using the specific chromogenic substrate S2302 (2 mM) in the presence of 50 mM Tris-HCl, pH 7.5 (90 μL). The kinetics of *p*-nitroaniline formation were monitored as described above. The amounts of plasma derived kallikrein generated by LOBE was estimated using a calibration curve made with known concentrations of purified plasma kallikrein and thus expressed as pmol of equivalent kallikrein/mL/min. Results represent the mean ± standard error of three independent experiments. In additional assays LOBE was incubated with human plasma in the presence of aprotinin (0.01, 1, 10, 1000 and 10000 KIU/mL) or anti-lonomic serum (1, 2, 10, 20 and 50%). Then, generated kallikrein activity was measured. To exclude the participation of procoagulant factors another set of experiments were done in a similar way that described above, but using a prothrombin/factor X deficient plasma (for details see supplementary methods, S1 Methods).

#### Vascular smooth muscle cell (VSMC) culture

A7r5 (ATCC^®^ CRL-1444) vascular smooth muscle cells (VMSC), obtained from rat thoracic aorta, were cultured in DMEM medium (high glucose) containing 10% FCS, 50 U/mL penicillin and 100 μg/mL streptomycin. The cultures were maintained at 37°C in humidified atmosphere containing 5% carbon dioxide (CO_2_). The cells were passaged at confluence following dissociation with trypsin/EDTA (0.05% / 0.01%), seeded into new T75 culture flasks and used from passages 3–14.

#### *L*. *obliqua* venom–induced kallikrein activation on VSMC

Aliquots of 5 x 10^3^ VSMC were seeded in 96-well plates overnight in a DMEM medium containing 10% FCS. The cells were washed twice in PBS, the medium was replaced by DMEM containing 1% FCS and VSMC were activated in the presence of different concentrations of LOBE (1, 10, 100 and 200 μg/mL). After 24 h incubation at 37°C in a 5% CO_2_ atmosphere, the cells were washed in PBS, incubated with human plasma (diluted 1:10) for 10 min and generated kallikrein was estimated by the addition of S2302 (2 mM). The kinetics of *p*-nitroaniline formation were monitored (405 nm) at a time interval of 14 s during a total time of 30 min on the microplate reader spectrophotometer (SpectraMAX 190, Molecular Devices, Sunnyvale, CA, USA). In another set of experiments, VSMC culture were treated (24 h) with aprotinin (1, 10, 100, 500 KIU/mL) and washed twice in PBS prior LOBE (100 μg/mL) treatment for additional 6 h. After a second step wash, VSMC were incubated with diluted human plasma and kallikrein formation was monitored as described above.

#### VSMC surface—Procoagulant assay

Procoagulant activity was measured on the surface of VSMC monolayers (5 x 10^3^) cultured in DMEM containing 10% FCS and seeded in 96-well plates overnight. For the experiment, cells were washed twice in PBS, the medium was replaced by DMEM containing 1% FCS and VSMC were incubated with plasma derived from control rats (animals injected with PBS) or plasma from envenomed rats (animals injected with 1.5 mg/kg LOBE, via s.c—for details related to the *in vivo* envenomation protocol, see below). After 24 h incubation at 37°C in a 5% CO_2_ atmosphere, the cells were washed twice in PBS, incubated with normal rat plasma (50 μL) for 10 min and coagulation time was measured in the presence (100 μL) of 20 mM Tris-HCl, pH 7.5 containing 10 mM CaCl_2_. The kinetics of clot formation was monitored (650 nm) at a time interval of 10 s during a total time of 45 min on the microplate reader spectrophotometer (SpectraMAX 190, Molecular Devices, Sunnyvale, CA, USA). In another set of experiments, VSMC culture were treated (24 h) with aprotinin (100 KIU/mL), washed twice and then treated with envenomed or control plasma (10%) for additional 6 h. After a second step wash, VSMC were incubated with normal rat plasma and coagulation time was determined as described above.

#### Cell migration

Cell migration was evaluated by wound healing assay **[27]**. Briefly, VSMC on the 96-well culture plates were incubated approximately 80% confluent and then made a scratch with yellow tip. Next, VSMC were treated with LOBE (1, 10 and 50 μg/mL) in DMEM containing 1% FCS and incubated at 37°C in a 5% CO_2_ atmosphere for 24 h. Microscopic photographs were captured at the beginning (0 h) and after 24 h using a Nikon Eclipse TE 2000-U optical microscope. Clear areas were measured and compared using Image J software (https://imagej.nih.gov/ij/) and the results were expressed as percent values of wound repaired area. Alternatively, VSMC were treated (2–30 μL) with diluted plasma (1:10) from control rats (animals injected with PBS) or plasma from envenomed rats (animals injected with 1.5 mg/kg LOBE, via s.c—for details related to the *in vivo* envenomation protocol, see below). In another set of experiments, VSMC culture were treated (24 h) with aprotinin (100 KIU/mL) prior to the initial scratch. Next, VSMC were washed twice in PBS and treated with LOBE (10 μg/mL) or envenomed diluted plasma (10 μL). In all experiments, cell migration was evaluated 24 h after the initial scratch as described above.

#### Cell proliferation

Proliferation of VSMC were evaluated by the MTT (3-(4,5-Dimethylthiazol-2-yl)-2,5-diphenyltetrazolium bromide) assay. Cells were seeded overnight in 96-well plates at 1 x 10^4^ cells/well using a DMEM medium containing 10% FCS. The cells were washed twice in PBS, medium was replaced by DMEM containing 1% FCS and cultures were treated or not with aprotinin (100 KIU/mL) 24 h prior to the addition of LOBE (10 μg/mL) or plasma derived from envenomed rats (10 μL of diluted plasma 1:10). After 24 h incubation at 37°C in a 5% CO_2_ atmosphere, medium and treatments were removed and MTT (0.5 mg/mL) solution was added to each well followed by incubation for 4 h. Next, formazan crystals were solubilized in dimethyl sulfoxide (100 μL) and the absorbance was measured at 565 nm. The results were expressed as fold increase to control (cells treated with PBS or plasma derived from non-envenomed animals—control plasma).

#### Intracellular reactive oxygen species (ROS) production

Intracellular ROS production in A7r5 VSMC cells were monitored as described in **[22]**. 5 x 10^3^ cells/well were seeded in 96-well black plates overnight in DMEM medium containing 10% FCS. The cells were washed twice in PBS and the medium was replaced by DMEM containing 1% FCS for 1 h. After this time the cells were loaded with DCF (10 μM) for 1h and washed twice in PBS to remove free probe. Next, VSMC were stimulated with LOBE (10–50 μg/mL) or diluted plasma (1:10) derived from envenomed rats (0.1–10%) in Hank’s Buffered Salt Solution (0.137 M NaCl, 5.4 mM KCl, 0.25 mM Na_2_HPO_4_, 0.44 mM KH_2_PO_4_, 1.3 mM CaCl_2_, 1.0 mM MgSO_4_, and 4.2 mM NaHCO_3_, pH 7.2). Generated ROS was monitored by CM-H2DCFDA fluorescence at excitation and emission wavelengths of 495 and 530 nm, respectively, using a SpectraMAX M3 microplate reader (Molecular Devices, Sunnyvale, CA, USA). Alternatively, VSMC were treated or not with aprotinin (100 KIU/mL) for 24 h prior to DCF loading and stimulation with LOBE (10 μg/mL) or plasma from envenomed rats (2 or 10%). Then, CM-H2DCFDA fluorescence was evaluated as described above.

### *In vivo* studies

#### Animals

Adult male Wistar rats, weighing 250–300 g, were supplied by the central animal facility of our institution. The animals were housed under standard conditions within a temperature controlled room (22–23°C, on a 12 h light/dark cycle, with the lights on at 7:00 am), and had free access to water and food.

#### Ethics statement

All the procedures involving animals were carried out in accordance with the Guiding Principles for the Use of Animals in Toxicology (International Society of Toxicology, http://www.toxicology.org) and the Brazilian College of Animal Experimentation (COBEA). The experimental protocol was approved by the ethical committee on research animal care of the Federal University of Rio Grande do Sul, Brazil (register number **2008177/2009**) and by the Institute's Animal Ethics Committee of the Experimental Research Center at Clinical Hospital, Porto Alegre, RS, Brazil (protocol number **16–054**).

#### Venom induced-acute kidney injury (AKI)

The time course of alterations in KKS components during *L*. *obliqua* induced-AKI was examined using an *in vivo* experimental model of envenomation. The animals were divided into one of two groups: A control group (CTRL), which contained animals (*n* = 6 per sampling time) that were injected subcutaneously (s.c) with 100 μL of a sterile PBS solution, and an experimental group (LOBE), which contained animals (*n* = 6 per sampling time) that were injected s.c with a solution containing 1.5 mg of the LOBE per kg of body weight in a final volume of 100 μL. At several time points post-venom injection (12, 24, 48 and 96 h), blood and kidney samples were obtained for plasma pre-kallikrein and renal kallikrein measurements. The venom dose and times post-envenomation were selected based on the results of previous experiments that were conducted using rats as an animal model **[6]** to reproduce the kidney damage and consumption coagulopathy that has been observed in humans **[3,25,28,29]**.

#### Pharmacological treatments

A pharmacological treatment based on kallikrein systemic inhibition was designed to study its roles in *L*. *obliqua* venom–induced AKI. For this reason, animals were divided into four groups (*n* = 6/group): (*i*) Control group (CTRL), in which the animals were treated intraperitoneally (i.p) with 250 μL of a sterile PBS solution 30 min prior to the s.c injection of 100 μL of a sterile PBS solution; (*ii*) LOBE + PBS, in which the animals were treated with 250 μL of PBS (i.p) 30 min prior to the injection of the LOBE (1.5 mg/kg, s.c); (*iii*) LOBE + APROTININ, in which the animals were treated with the kallikrein inhibitor, aprotinin (5.6 mg/kg, i.v, corresponding to 40,000 kallikrein inhibitory units/kg, diluted in sterile saline), 30 min prior to the injection of the LOBE (1.5 mg/kg, sc); and (*iv*) APROTININ + PBS, in which the animals were treated with 250 μL of PBS (i.p) 30 min prior to the injection of aprotinin (40,000 KIU/kg, i.v). Immediately after the treatments, the animals were distributed individually in metabolic cages. At 24 h post-venom injection, blood, urine and kidney samples were obtained for biochemical, histopathological, kallikrein and renal tissue factor activity analysis. Aprotinin doses used here were selected based on previously published data **[8,21,30]**.

#### Blood, urine and kidney samples

Blood samples were obtained by cardiac puncture in rats that had been anesthetized with ketamine (75 mg/kg) and xylazine (10 mg/kg) and anticoagulated with 1:10 (v/v) 3.8% trisodium citrate. Plasma was obtained by centrifugation at 1500 x g for 10 min and stored at– 80°C prior to the biochemical determinations. Urine samples were collected directly from the metabolic cages and centrifuged at 2500 x g for 5 min. The supernatants were stored under the same conditions used for the plasma samples. After blood collection, the hearts were perfused through the left ventricle with a PBS solution, and a circulatory circuit was opened by an incision in the right atrium to ensure the elimination of intravascular blood. Immediately after perfusion, the kidneys were quickly removed. One of the kidneys was fixed for histological analysis, and the second was frozen in liquid nitrogen and stored at– 80°C prior to the measurements of tissue factor and kallikrein activities, nitrate/nitrite, superoxide anion, GSH and pro-inflammatory cytokine levels. For the measurements of tissue factor and kallikrein activities and nitrate/nitrite levels, a portion of the kidney was homogenized in a cold PBS solution containing 1% Triton X-100 and centrifuged at 9500 x g for 15 min. The supernatants were immediately used in the assays.

#### Analysis of renal function

The levels of creatinine, urinary γ-glutamyl transferase (γ-GT) activity and urinary proteins were used for renal function assessments. Urine and plasma creatinine, urinary γ-GT activity and proteinuria were determined spectrophotometrically (SP-220 BioSpectro Spectrophotometer, Paraná, Brazil) using commercially available kits (BioClin/Quibasa, Belo Horizonte, Brazil). The glomerular filtration rate (GFR), expressed as mL/min/100 g of body weight, was estimated by the creatinine clearance (C_cr_) using the standard formula: C_cr_ = U_cr_.V/P_cr_, where U_cr_ is the urinary creatinine concentration, V is the urinary output and P_cr_ is the plasma creatinine concentration. Water fractional excretion (FE_H2O_), expressed as a %, was determined using the equation: FE_H2O_ = V/GFR.100. Sodium (FE_Na_^+^), potassium (FE_K_^+^) and chloride (FE_Cl-_) fractional excretion (expressed as %) were calculated according to the equation: FE = UE/PF.100. UE represents the urinary excretion of each ion and PF is the amount filtered in plasma (both expressed as nmol/min).

#### Renal tissue factor activities

Renal tissue factor (TF) was measured as described in **[6]**. Samples from kidney extracts (10 μg) were incubated with a concentrated mixture of clotting factors (FVII, FIX and FX, 15 μg) in 20 mM Tris-HCl, pH 7.4, containing 10 mM CaCl_2_ for 10 min at 37°C. As kidney extracts are a source of TF, activated factor Xa (FXa) that was produced during the reactions can be used as an indirect index of TF in these samples. Thus, FXa activity was detected by the addition of a specific chromogenic substrate (0.2 mM S2222). The kinetics of *p*-nitroaniline release were monitored at 405 nm for 30 min using a microplate reader spectrophotometer (SpectraMAX, Molecular Devices Co., Sunnyvale, USA). Results were expressed as μmol of FXa generated per min per mg of kidney tissue.

#### Plasma prekallikrein and renal kallikrein activities and expression

To measure plasma prekallikrein, plasma samples (5 μL) were previously incubated with 50 mM Tris-HCl, pH 7.4, containing 100 μM ellagic acid, phospholipids, 0.005% bovine albumin and 10 mM CaCl_2_ for 10 min at 37°C. Activated kallikrein that was produced during the reaction was detected by the addition of 0.2 mM S2302. To measure renal kallikrein, kidney extracts were prepared as described above, diluted (1:2) and incubated (15 μL) in 50 mM Tris-HCl, pH 7.4, for 10 min at 37°C. Kallikrein activity was detected by the addition of 0.2 mM S2366. In both cases, the kinetics of *p*-nitroaniline release were monitored at 405 nm for 30 min in a final volume of 100 μL using a microplate reader spectrophotometer (SpectraMAX, Molecular Devices Co., Sunnyvale, USA). Each sample was measured in triplicate, and the results were expressed as μmol of *p*-nitroaniline released per min per mg of protein. Renal kallikrein expression was detected by immunoblotting using a rabbit anti-rat KLK-1 polyclonal antibody (NSJ bioreagents, San Diego, CA, USA) raised against tissue kallikrein-1. Kidney extracts (30 μg) were separated by SDS-PAGE 12% under reducing conditions, transferred onto nitrocellulose membranes, incubated with primary and secondary-horseradish peroxidase conjugated antibody and revealed using the colorimetric kit Opti-4CN (Bio-Rad, Hércules, CA, USA). Protein expression levels was normalized with β-actin and quantified using Image J software.

#### Nitric oxide determinations

Total nitrate and nitrite kidney levels were determined as an indication of nitric oxide (NO) production **[31]**. Kidney samples (100 μL) were deproteinized by the addition of 1:1 (v/v) ethanol and incubated overnight at 4°C. After centrifugation at 1500 X g for 10 min, the supernatants (100 μL) were mixed with 8 mg/mL of VCl_3_ (100 μL) in 96 well plates. The Griess reagent (100 μL of 2% SULF + 0.1% NEED) was immediately added to the wells, and the absorbance was then determined at 540 nm after 30 min incubation at room temperature protected from light. Sample absorbance values were compared with a standard curve made with known concentrations of sodium nitrate (1–200 μM).

#### Reduced glutathione (GSH) and superoxide anion levels

GSH levels were determined using a spectrophotometric method **[32]**. Kidney homogenates (0.5 mL) were diluted in distilled water (1 mL) and treated with trichloroacetic acid (0.5 mL, 50% weight/volume). After 15 min, the homogenates were centrifuged at 1500g for 15 min, and the supernatant (1 mL) was mixed with 2 mL of a solution containing Tris 0.4 M (pH 8.9) containing 50 μL of DTNB (5,5-dithio-bis-(2-nitrobenzoic acid)). After 5 min, the measurements were performed at 412 nm and the results are expressed as nmol of GSH per mg of tissue using a standard GSH curve (0.09–100 nmol). The superoxide anion production was estimated in kidney homogenates based on NBT (Nitroblue Tetrazolium) reduction assay **[33]**. Briefly, the homogenates (50 μL) were mixed with 0.1% NBT solution (100 μL) and incubated in 96-well plates at 37°C for 1 h. The aqueous mixture was removed carefully from the wells, and the formazan precipitate was solubilized by adding 2M KOH (120 μL) and dimetil sulfoxide (140 μL) solutions in each well. The absorbance was measured at 600 nm and the results are expressed as NBT reduction (OD at 600 nm).

#### Histology

For the renal histopathological analysis, the kidneys were collected, as described previously, sectioned sagitally, and fixed in 10% buffered formaldehyde, pH 7.2. After processing in alcohol and xylol, the organs were embedded in paraffin, and 4 μm thick sections were obtained and stained with hematoxylin and eosin (H&E) or periodic acid Schiff (PAS). Thirty high-power fields (40 X) of the renal cortex were randomly selected for each section, and the presence of the following alterations was observed: Intratubular casts, tubular degeneration and cellular inflammatory infiltrate (renal inflammation). A semi-quantitative score (ranging from 0 to 4) was attributed to each of these alterations as follows: a score of zero was attributed to normal regions, a score of 1 was attributed to changes affecting less than 20% of the region under examination, a score of 2 was attributed to changes affecting 20 to 40% of the region, a score of 3 was attributed to changes affecting 40 to 80% of the region and a score of 4 was attributed to changes affecting greater than 80% of the region. All scoring was performed in a blinded manner.

#### Assessments of renal pro-inflammatory cytokine levels

The levels of pro-inflammatory cytokines (interleukin [IL] 1-β and tumor necrosis factor [TNF]-α) were determined in the kidney supernatants (homogenized as described above) using enzyme-linked immunosorbent assay (ELISA) kits from R&D Systems (Minneapolis, MN, USA), according to the manufacturer's recommendations.

#### Renal N-acetyl-β-d-glucosaminidase (NAG), myeloperoxidase (MPO) and matrix metalloproteinase (MMP) activities

NAG and MPO activities were used as markers of kidney macrophage and neutrophil recruitment, respectively **[34]**. Kidney tissue samples (~ 100 mg) were homogenized in 50 mM K_2_HPO_4_, pH 6.0 containing HTAB (0.5% w/v), and the homogenates were centrifuged (16000 x g) for 5 min. For NAG assay, the supernatants (20 μL), were added to 96-well plates and incubated in 50 mM K_2_HPO_4_, pH 6.0 (80 μL). The enzymatic reaction was initiated by the addition of 4-nitrophenyl N-acetyl-β-d-glucosaminide substrate (2.24 mM). After incubation (10 min at 37°C) the reaction was stopped by the addition of 0.2 M glycine, pH 10 (100 μL). The enzymatic activity was determined spectrophotometrically at 400 nm and results were expressed as NAG activity (optical density/min/mg kidney tissue). For MPO assay, the supernatants (30 μL) obtained from kidney homogenates were incubated in 50 mM K_2_HPO_4_, pH 6.0 (200 μL) containing *o*-dianisidine dihydrochloride (0.0167%, w/v) and hydrogen peroxide (0.05%, v/v). The absorbance was then determined after 5 min at 450 nm. The results of MPO activity were also expressed as optical density/min/mg kidney tissue. MMP activity was estimated by gelatin zymography. Kidney extracts (30 μg) were separated by an SDS-polyacrylamide gel electrophoresis (12%) copolymerized with 1% gelatin under non-reducing conditions. After SDS removal in 1% triton X-100, clear bands were observed in coomassie-blue stained gels at 92 kDa (MMP-9) and 72 kDa (MMP-2). Images were analysed and quantified using Image J software.

### Statistical analysis

The data are presented as the means ± SE, and significant differences were analyzed using one-way ANOVA followed by an unpaired t-test with a Bonferroni correction for multiple comparisons. P-values of 0.05 were considered to be significant. Statistical analyses were performed using GraphPad Prism (GraphPad Software Inc., San Diego, CA, USA).

## Results

### Kallikrein is generated directly by *L*. *obliqua* venom and indirectly by venom-activated vascular smooth muscle cells (VSMC)

Kallikrein is a key enzyme in the kinin system, but also can activate factor XII triggering the blood coagulation intrinsic cascade. As it is known that *L*. *obliqua* venom activates coagulation factors (mainly factor X and prothrombin) **[9]**, we first investigated whether the venom could directly activate plasma prekallikrein *in vitro*.

**Fig 1:**
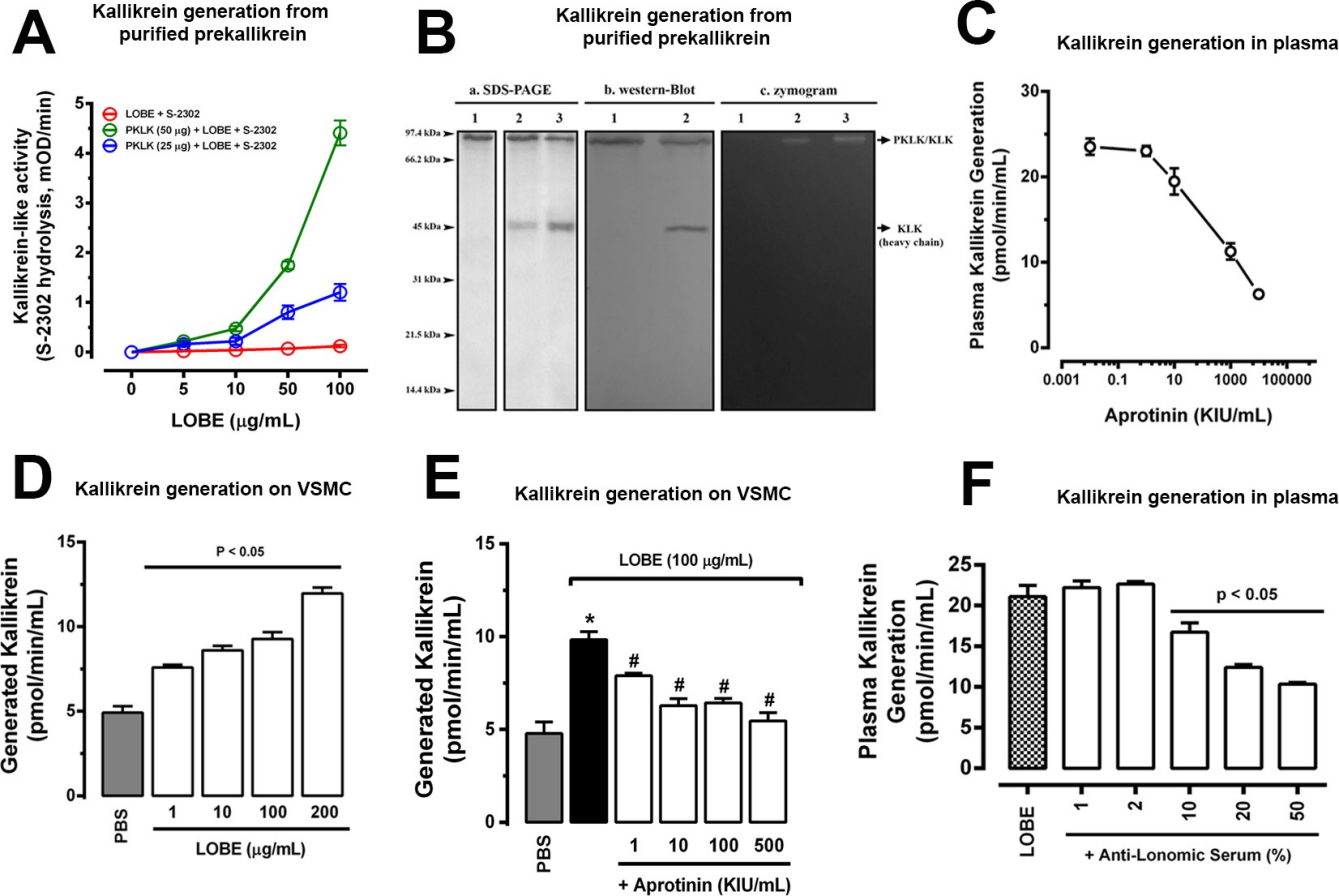
*L*. *obliqua* venom-induced prekallikrein activation. **A.** Different concentrations of *L*. *obliqua* bristle extract (LOBE) was incubated in the presence or absence of purified plasma prekallikrein (PKLK) and generated kallikrein (KLK) was then measured by the addition of S-2302. **B.** LOBE-induced PKLK activation was confirmed by SDS-PAGE, western-blot and zymogram gels. a. SDS-PAGE: 1 –purified plasma PKLK (25 μg); 2 –Purified PKLK (25 μg) previously incubated with LOBE (5 μg) during 15 min; 3—Purified PKLK (25 μg) previously incubated with LOBE (5 μg) during 30 min. All samples were running in 12% gels under reducing conditions. b. western-blot: 1 –Purified PKLK (25 μg); 2—Purified PKLK (25 μg) previously incubated with LOBE (5 μg) during 30 min. In both cases, samples were running under reducing conditions and plasma PKLK or KLK heavy chain were identified using a specific anti-plasma PKLK antibody. c. zymogram. 1 –purified plasma PKLK (25 μg); 2 –Purified PKLK (25 μg) previously incubated with LOBE (5 μg) during 15 min; 3—Purified PKLK (25 μg) previously incubated with LOBE (5 μg) during 30 min. The enzymatic activity of generated KLK was detected by SDS-PAGE 12% containing a mixture of casein + plasminogen under non-reducing conditions. **C.** Human plasma was incubated with different concentrations of aprotinin and kallikrein generation was triggered by the addition of LOBE (100 μg/mL). **D.** VSMC culture were treated with different concentrations of LOBE for 24h and washed twice in PBS. Then the monolayers were incubated with human plasma and contact system activation was monitored through kallikrein formation. **E.** VSMC culture were treated (24h) with different concentrations of aprotinin, washed twice, and treated with LOBE (100 μg/mL) for 6h. After a second step wash, VSMC were incubated with human plasma and kallikrein formation was monitored. **F.** Human plasma was incubated with different concentrations of anti-lonomic serum (anti-venom produced by the Butantan Institute in Brazil) and kallikrein generation was triggered by the addition of LOBE (100 μg/mL). In all experiments the kinetics of kallikrein generation was monitored using the chromogenic substrate S-2302. Data represents mean of three independent experiments ± SE. * denotes p<0.05 vs PBS group and ^#^ denotes p<0.05 vs group treated with LOBE (100 μg/mL). KIU denotes kallikrein inhibitory units.

For that, prekallikrein was purified from rat plasma using classical procedures of protein precipitation and chromatographic steps, which gave us a relatively homogeneous preparation to be further used in venom-induced prekallikrein activation assays **(S1 Fig)**. The identity of plasma prekallikrein in our preparation was also confirmed by immunoblot **(S1 Fig)**. *In vitro* incubation of *L*. *obliqua* bristle extract (LOBE) with prekallikrein induced a dose-dependent kallikrein-like activity which was significantly higher than the intrinsic LOBE activity upon S2302 synthetic substrate **(Fig 1A)**. In a different way, LOBE activity upon S2222 or S2238 substrates were almost equal in the presence or absence of plasma prekallikrein, confirming that our preparation was free from procoagulant zymogens (FX and prothrombin), since there is not FXa or thrombin-generating activity **(S2 Fig)**.

Prekallikrein migrates on SDS-PAGE as a single band about of 88 to 90 kDa under reduced conditions. After incubation with LOBE for 15 or 30 min an additional band appeared around of 40–45 kDa under reduced conditions, which probably correspond to the heavy chain of active kallikrein **(Fig 1B)**. This was confirmed by western-blot using a specific anti-rat prekallikrein antibody **(Fig 1B)**. In fact, the difference between prekallikrein and active kallikrein is only a single disulfide bond cleavage. Thus, under non-reduced conditions active kallikrein migrates as the same single band as prekallikrein and under reduced conditions the heavy and light chains of kallikrein can appeared, been mainly the light chain more susceptible to autolysis and degradation **[35]**. We also confirmed that LOBE can generate active kallikrein from prekallikrein using a substrate gel electrophoresis copolymerized with casein and plasminogen (zymogram) **(Fig 1B)**. In this case, active kallikrein is able to produce plasmin from plasminogen leading to casein degradation and formation of a clear band around of 88–90 kDa. Since the gel was running under non-reduced conditions and it is well-known that kallikrein generate plasmin **[26]**, the band in **Fig 1B** is probably active kallikrein.

Experiments were also done to verify LOBE-induced kallikrein activation in human plasma. In the absence of cofactors (such as calcium or phospholipids), LOBE appears to be able to generate active kallikrein after incubation with human normal plasma **(S3 Fig)**. Previous incubation of plasma with aprotinin, a kallikrein inhibitor, dose-dependently reduced this effect **(Fig 1C)**. Another set of assays were designed using prothrombin and factor X-deficient plasma (plasma -PThr/-FX) to completely eliminate venom FX- and prothrombin-activating activities interference. As depicted in **S4 Fig**, plasma -PThr/-FX was free from FXa or thrombin-like activities after ellagic acid incubation **(S4A Fig)**, but displayed kallikrein-like activity after incubation with LOBE. kallikrein-like activity generated in plasma -PThr/-FX was higher than the direct effect of LOBE on S-2302 substrate and was significantly inhibited by aprotinin **(S4B Fig)**. However, the activity upon S2222 and S2238 hydrolysis was the same in presence or absence of plasma -PThr/-FX, indicating that there was no thrombin or FXa being generated during these reactions **(S4C and S4D Fig)**, which only occurred when a normal plasma was incubated with LOBE **(S4E Fig)**.

Since *L*. *obliqua* venom was previously shown to cause vascular alterations by inducing proliferation and migration of VSMC **[22]**, we examined if the surface of VSMC previously activated with the venom could also participate generating active kallikrein in an indirect manner. In fact, vascular cell surface previously activated with LOBE is able to induce a significant increase in kallikrein formation in human plasma when compared to VSMC previously treated with PBS **(Fig 1D)**. When aprotinin was incubated with VSMC before LOBE-induced cell activation, there was also a decrease in plasma kallikrein formation on VSMC surface **(Fig 1E)**. Similarly to aprotinin the pre-treatment of human plasma with anti-lonomic serum also reduced venom-induced direct kallikrein formation **(Fig 1F)**.

### Kallikrein is generated *in vivo* during experimental envenomation and its activity is related to the coagulation disturbances

**Fig 2:**
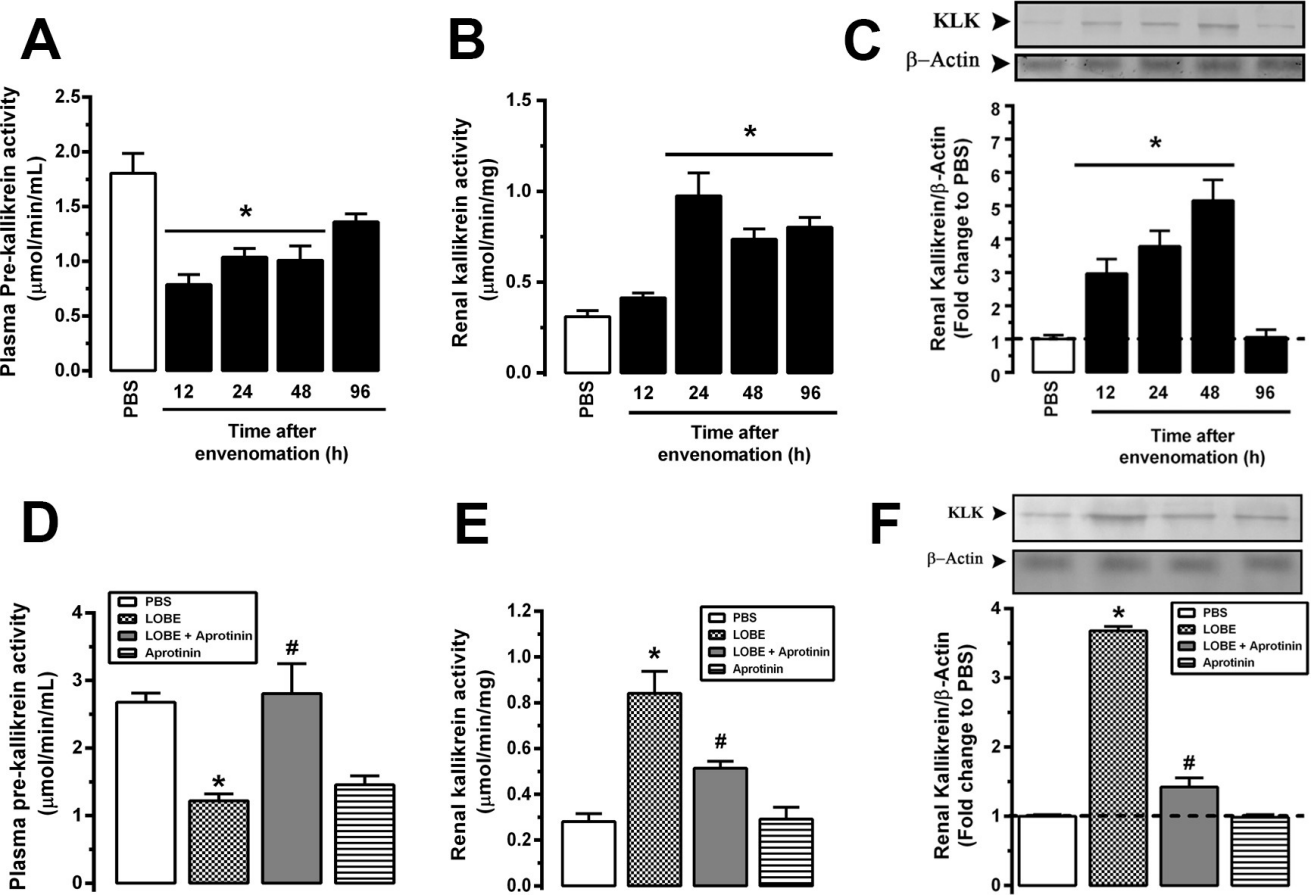
Kallikrein is generated during *L*. *obliqua* envenomation *in vivo*. Wistar rats were injected with LOBE (1.5 mg/kg, s.c) and the following parameters were determined at different times post-envenomation: **A.** Plasma prekallikrein levels. **B.** Renal kallikrein activity. **C.** Protein levels of renal kallikrein determined by western-blot using specific antibody against tissue kallikrein. In another set of experiments, groups of animals were treated with PBS, LOBE (1.5 mg/kg, s.c), aprotinin (40,000 KIU/mg, i.v) or received aprotinin (40,000 KIU/mg, i.v) 30 min prior to the injection of the LOBE (1.5 mg/kg, sc). After 24h the same parameters were determined: **D.** Plasma prekallikrein levels. **E.** Renal kallikrein activity. **F.** Protein levels of renal tissue kallikrein. Data represents mean of three independent experiments ± SE. * denotes p<0.05 vs PBS group and ^#^ denotes p<0.05 vs group treated with LOBE.

*In vivo*, *L*. *obliqua* envenomation is characterized by a consumption coagulopathy followed by blood incoagulability **[7,36]**. As the *in vitro* results suggested the presence of a strong kallikrein activating activity in the venom, we then decide to study the kinetics of kallikrein generation *in vivo* in rats subcutaneously injected with LOBE (1.5 mg/kg). As depicted in **Fig 2A** plasma prekallikrein activity decreased in envenomed animals, suggesting that active kallikrein has been generated systemically during envenomation. Consistent with this observation, renal kallikrein activity significantly increased between 24 and 96 h post-venom injection **(Fig 2B)**. Also, tissue renal kallikrein expression increased at protein level as demonstrated by immunoblot, indicating that the KKS is activated in the kidneys of envenomed rats **(Fig 2C)**.

**Fig 3:**
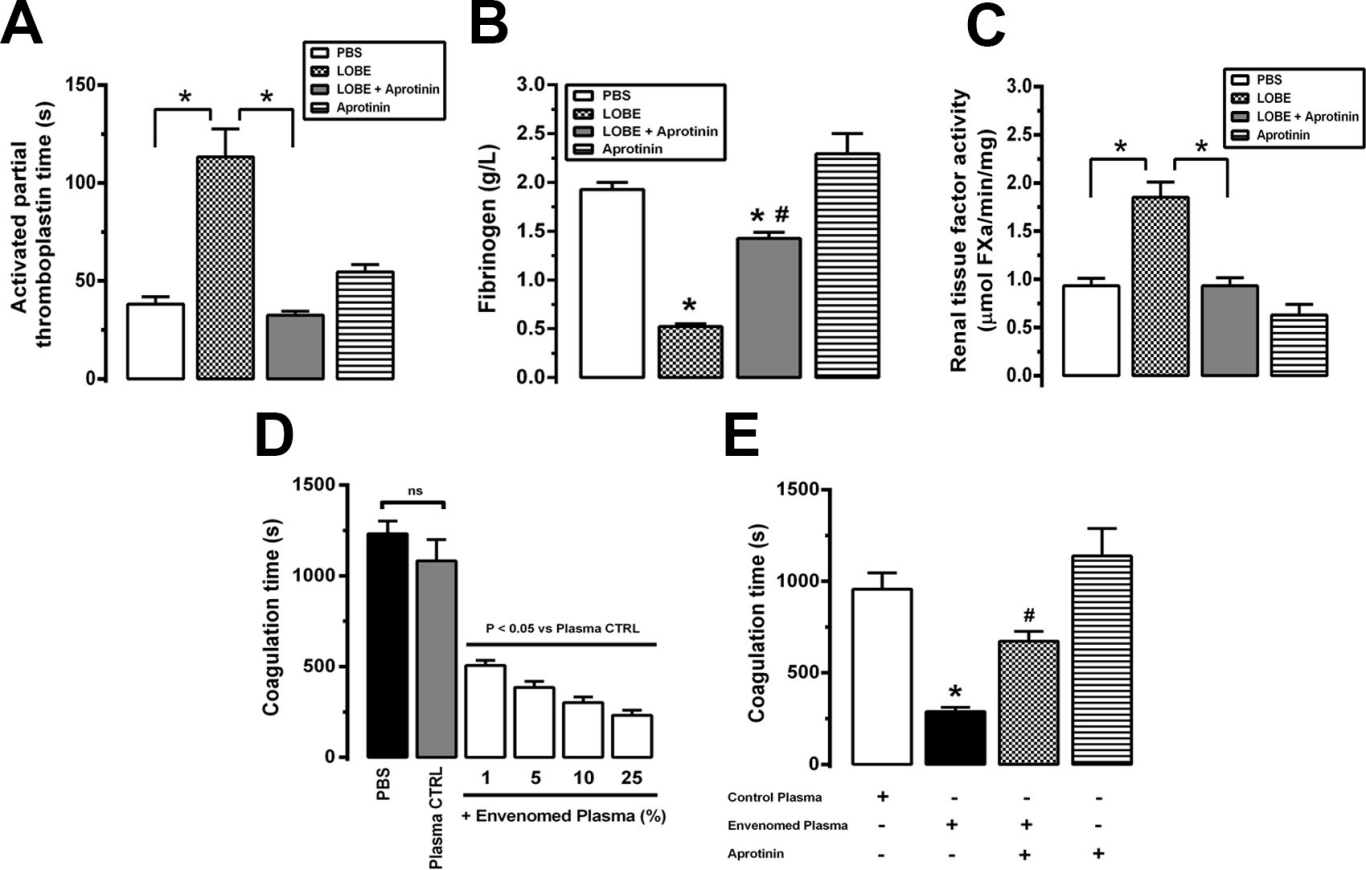
Kallikrein generated during *L*. *obliqua* envenomation *in vivo* is directly involved in venom-induced coagulation disturbances. Wistar rats were treated with PBS, LOBE (1.5 mg/kg, s.c), aprotinin (40,000 KIU/mg, i.v) or received aprotinin (40,000 KIU/mg, i.v) 30 min prior to the injection of LOBE (1.5 mg/kg, sc). After 24h the following renal parameters were determined: **A.** Activated partial thromboplastin time (APTT). **B.** Fibrinogen levels. **C.** Renal tissue factor levels. **D.** VSMC were cultured in the presence of plasma from control (animals treated with PBS) or envenomed rats. After 24h the monolayers were washed twice and coagulation time was determined by adding normal rat plasma in the presence of calcium ions. **E.** VSMC culture were treated (24h) with aprotinin (100 KIU/mL), washed twice and then treated with envenomed or control plasma (10%) for 6h. After a second step wash, VSMC were incubated with normal rat plasma and coagulation time was determined. Data represents mean ± SE. * denotes p<0.05 vs PBS group and ^#^ denotes p<0.05 vs group treated with LOBE or envenomed plasma; CTRL denotes control and ns denotes difference not significant.

To investigate the role of systemically generated kallikrein in the coagulation disturbances, Wistar rats were pre-treated intravenously with aprotinin (40,000 KIU/mg). Systemic kallikrein inhibition by aprotinin prevented plasma prekallikrein consumption **(Fig 2D)** and the consequent increase in tissue kallikrein activity and protein expression in the kidney of envenomed rats **(Fig 2E and 2F)**. Aprotinin also protected against blood incoagulability and consumption coagulopathy, restoring activated partial thromboplastin time **(Fig 3A)**, and recovering in 73% the fibrinogen levels **(Fig 3B)**. Furthermore, kidney tissue factor, a marker of procoagulant activity that is involved in intraglomerular fibrin deposition, was reduced in envenomed animals treated with aprotinin **(Fig 3C)**.

Next, we investigated whether the plasma obtained from envenomed animals would be able to activate VSMC leading cells to display a procoagulant effect. For this purpose, VSMC were cultured in the presence of plasma from control or envenomed rats. After 24 h the monolayers were washed twice to remove all plasma components and coagulation time was determined by adding normal fresh plasma in the presence of calcium ions to monitored clot formation. We found that even small quantities of envenomed plasma (such as 1% v/v) was able to activate and change VSMC to a procoagulant profile. There was a dose-dependent reduction in coagulation time when cells were pre-cultured with envenomed plasma comparing with cells cultured with plasma from control animals or PBS **(Fig 3D)** and this effect was partially attenuated in VSMC previously treated with aprotinin **(Fig 3E)**.

### Kallikrein inhibition ameliorates renal function during *L*. *obliqua* envenomation

**Fig 4:**
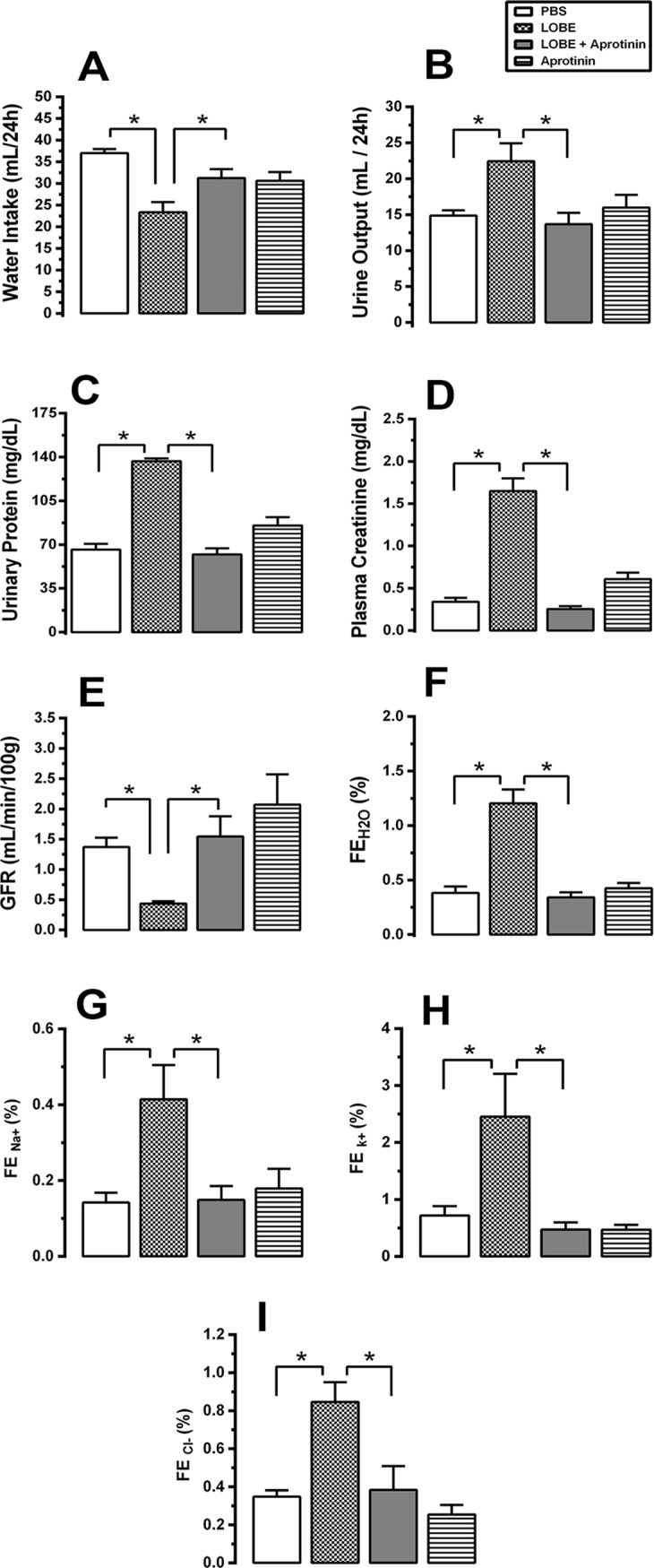
Kallikrein activation during *L*. *obliqua* envenomation plays a central role in renal functional impairment. Wistar rats were treated with PBS, LOBE (1.5 mg/kg, s.c), aprotinin (40,000 KIU/mg, i.v) or received aprotinin (40,000 KIU/mg, i.v) 30 min prior to the injection of LOBE (1.5 mg/kg, sc). After 24h the following renal parameters were determined: **A.** Water intake; **B.** Urine output; **C.** Urinary protein; **D.** Plasma creatinine; **E.** Glomerular filtration rate (GFR); **F.** Fractional excretion of water (FE_H2O_); **G.** Fractional excretion of sodium (FE_Na+_); **H.** Fractional excretion of potassium (FE_K+_) and **I.** Fractional excretion of chloride (FE_Cl-_). Data represents mean ± SE. * denotes p<0.05.

The results of a previous study indicated that *L*. *obliqua*–induced AKI in rats is accompanied by a sudden loss of glomerular filtration capacity, a decrease in tubular hydro-electrolytic transport and tubular necrosis. These alterations peaked between 12 and 48 h **[6]**. Here, we observed that after 24 h, rats injected with LOBE (1.5 mg/kg, s.c), presented a decrease in water intake and a significant polyuria, suggesting an increase in hydric balance (the ratio between urine output and water consumption) **(Fig 4A and 4B)**. These alterations were accompanied by increase in urinary protein excretion **(Fig 4C)**, plasma creatinine levels **(Fig 4D)** and a marked reduction in the glomerular filtration rate (GFR) **(Fig 4E)**. Tubular handling of water **(Fig 4F)** and electrolytes (Na^+^, K^+^ and Cl^-^) **(Fig 4G–4I)** were also impaired during envenomation. The pretreatment with aprotinin completely prevented these alterations. Systemic kallikrein inhibition restored fluid homeostasis and the tubular ability to retain water and electrolytes, as reflected by the fact that urinary output, hydric balance, FE_H2O_, FE_Na+_, FE_K+_ and FE_Cl-_ returned to the levels observed in the PBS-treated animals **(Fig 4A, 4B and 4F–4I)**. Aprotinin pretreatment also restored filtration capacity and protected the animals from venom-induced glomerular injury, as indicated by the levels of GFR, plasma creatinine and urinary protein, which had returned to the levels observed in the control animals **(Fig 4C–4E)**.

### Aprotinin protects against renal inflammation and morphological alterations during *L*. *obliqua* envenomation

**Fig 5:**
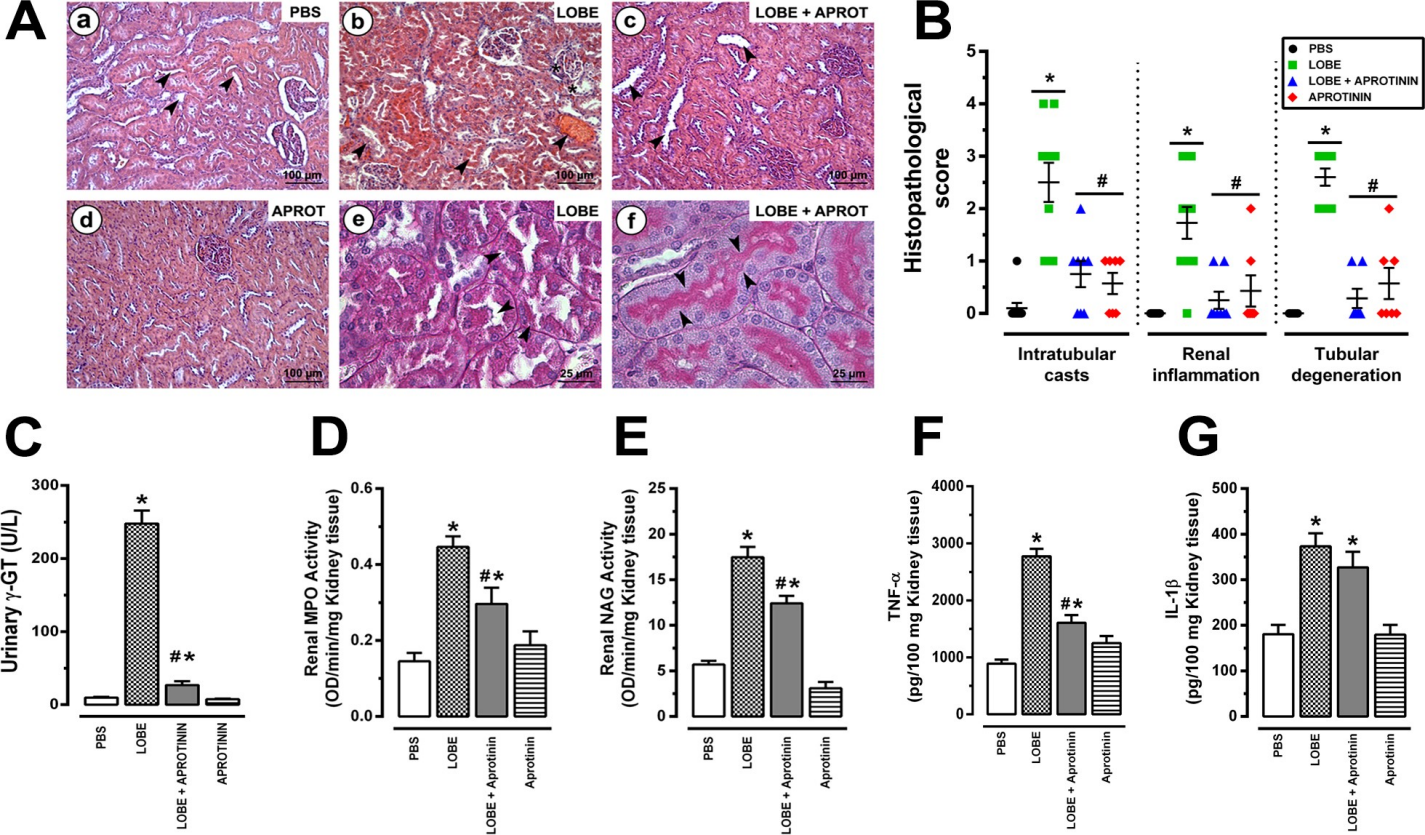
Effects of aprotinin on renal inflammation, tubular degeneration and morphological alterations induced by *L*. *obliqua* venom. **A.** Kidney morphological changes of Wistar rats pre-treated or not with aprotinin after 24h of envenomation. (a) cortical section stained with HE of an animal injected with PBS, showing normal glomerular and tubular (arrowheads) structures; (b) cortical section stained with HE of an animal injected with LOBE (1.5 mg/kg, s.c), showing acute tubular necrosis, intratubular and hematic casts (arrowheads); (c) cortical section stained with HE of an animal that received aprotinin (40,000 KIU/mg, i.v) 30 min prior to the injection of the LOBE (1.5 mg/kg, sc), showing normal glomerular and tubular structures and absence of protein or hematic deposits inside tubules (arrowheads); (d) cortical section stained with HE of an animal injected with aprotinin (40,000 KIU/mg, i.v), showing normal morphology; (e) detail of a cortical section stained with PAS from an envenomed rat, pointing out tubular degeneration and formation of necrotic cell deposits inside tubules (arrowheads); and (f) detail of a cortical section stained with PAS from an envenomed rat pre-treated with aprotinin, highlighting normal tubular morphology and brush border structures (arrowheads). **B.** Histopathological scores for all sections according to the presence of intratubular casts, renal inflammation and tubular degeneration (for details see Material and Methods); **C.** Levels of urinary γ-glutamyltransferase (an index of tubular injury); **D.** Levels of kidney myeloperoxidase (an index of neutrophil accumulation); **E.** Levels of kidney N-acetylglucosaminidase activity (an index of macrophage accumulation); **F.** Levels of renal tumor necrosis factor– α; and **G.** Levels of renal interleukin—1β. Data represents mean ± SE. * denotes p<0.05 vs PBS group and ^#^ denotes p<0.05 vs group treated with LOBE.

Our next step was to investigate whether aprotinin pretreatment would be effective in preventing the development of kidney lesions during *L*. *obliqua*-induced AKI. Along with the progressive decline in renal function, LOBE injection also induced progressive degenerative lesions that were compatible with acute tubular necrosis (ATN) **(Fig 5)**. Increased acidophilia, loss of the proximal brush border, inflammatory infiltration, edema, cytoplasmic vacuolation, degeneration and desquamation of necrotic cells forming intratubular casts were common alterations that were observed in envenomed animals (LOBE group). In general, pretreatment with aprotinin prevented the occurrence of these morphological alterations **(Fig 5A)**. Intratubular obstruction, which is caused by proximal brush border cell detachment to the tubule lumen, is almost prevented by kallikrein inhibition with aprotinin, as can be observed in PAS stained sections **(Fig 5A–5E and 5A–5F)**. Nevertheless, the histopathological scores for intratubular casts, tubular degeneration and renal inflammation were ameliorated following systemic kallikrein inhibition **(Fig 5B)**. Furthermore, confirming these histological observations, aprotinin reduced γ-glutamyltransferase (γ-GT) urinary activity **(Fig 5C)**, which is considered to be an efficient biomarker for ATN **[37]**. One of the mechanisms involved in the beneficial effects of kallikrein inhibition appeared to be the suppression of renal inflammation, because pretreatment with aprotinin partially reduced renal neutrophil and macrophage accumulation **(Fig 5D and 5E)**. Regarding pro-inflammatory cytokines, there was a significant decrease in renal tumor necrosis factor-α (TNF-α) **(Fig 5F)** whereas interleukin 1-β (IL-1β) levels tend to decrease, but did not reach statistical difference **(Fig 5G)**.

### Kallikrein is involved in nitric oxide (NO), matrix metalloproteinase (MMP) and reactive oxygen species (ROS) generation in kidney and VSMC during envenomation

**Fig 6:**
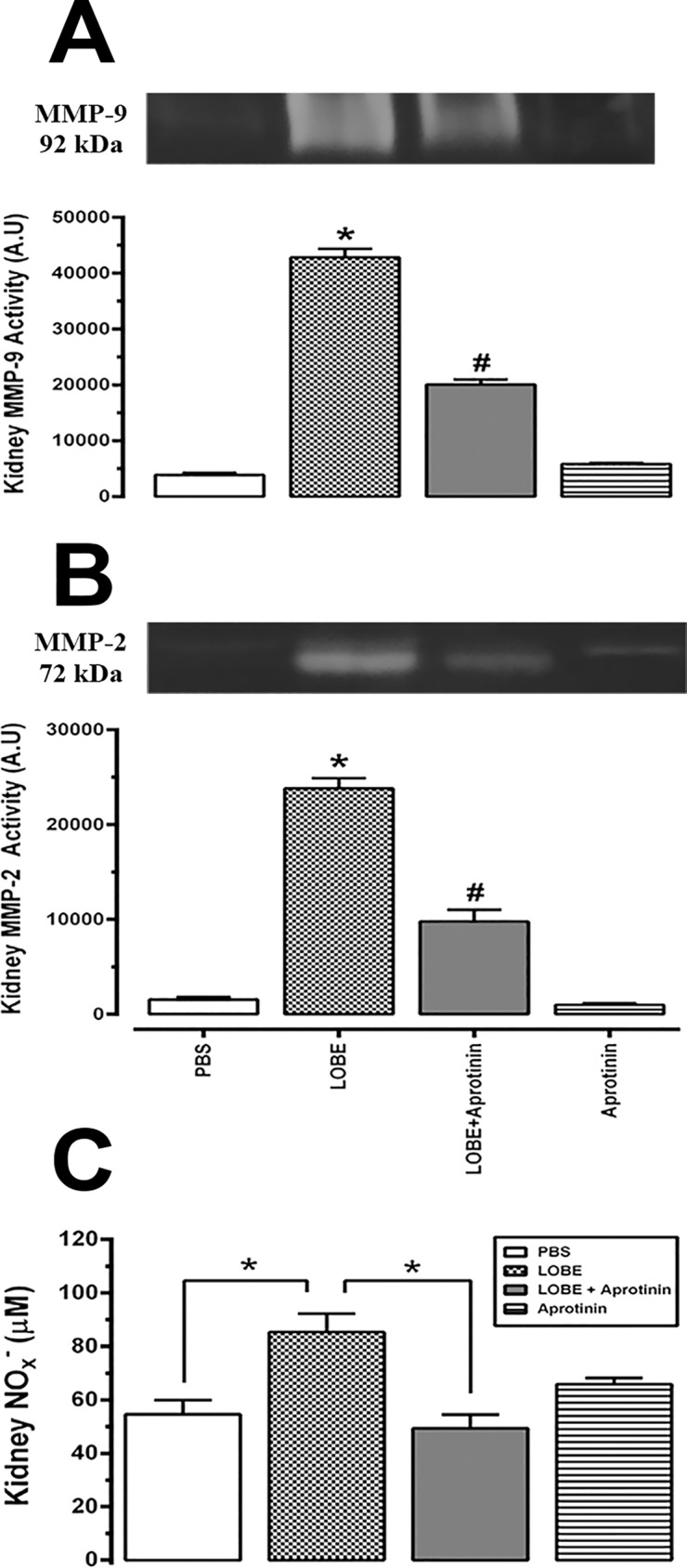
Kidney matrix-metalloproteinase (MMP) up-regulation and nitric oxide (NO) generation during *L*. *obliqua* envenomation: Effects of aprotinin treatment. Kidneys from Wistar rats pre-treated or not with aprotinin were collected after 24h of *L*. *obliqua* venom injection and MMP-9 **(A)** and MMP-2 **(B)** activities were measured by gelatin zymography. **C.** Kidney NO levels were estimated by the Griess method. Data represents mean ± SE. * denotes p<0.05 vs PBS group and ^#^ denotes p<0.05 vs group treated with LOBE.

To continue investigating the pro-inflammatory consequences of systemic kallikrein activation, MMP activity, NO and ROS production were determined **(Fig 6)**. LOBE leads to an increase of MMP-9 and MMP-2 activities in the kidney 24 h post-venom injection. Kallikrein systemic inhibition (with aprotinin) attenuated this effect by around of 50% **(Fig 6A and 6B)**. Likewise, as a consequence of kallikrein activation and probably bradykinin formation, nitrate/nitrite levels (which are indicative of NO production) increased in the kidney. Again, this effect was attenuated and returned to control levels in those animals pre-treated with aprotinin **(Fig 6C)**.

**Fig 7:**
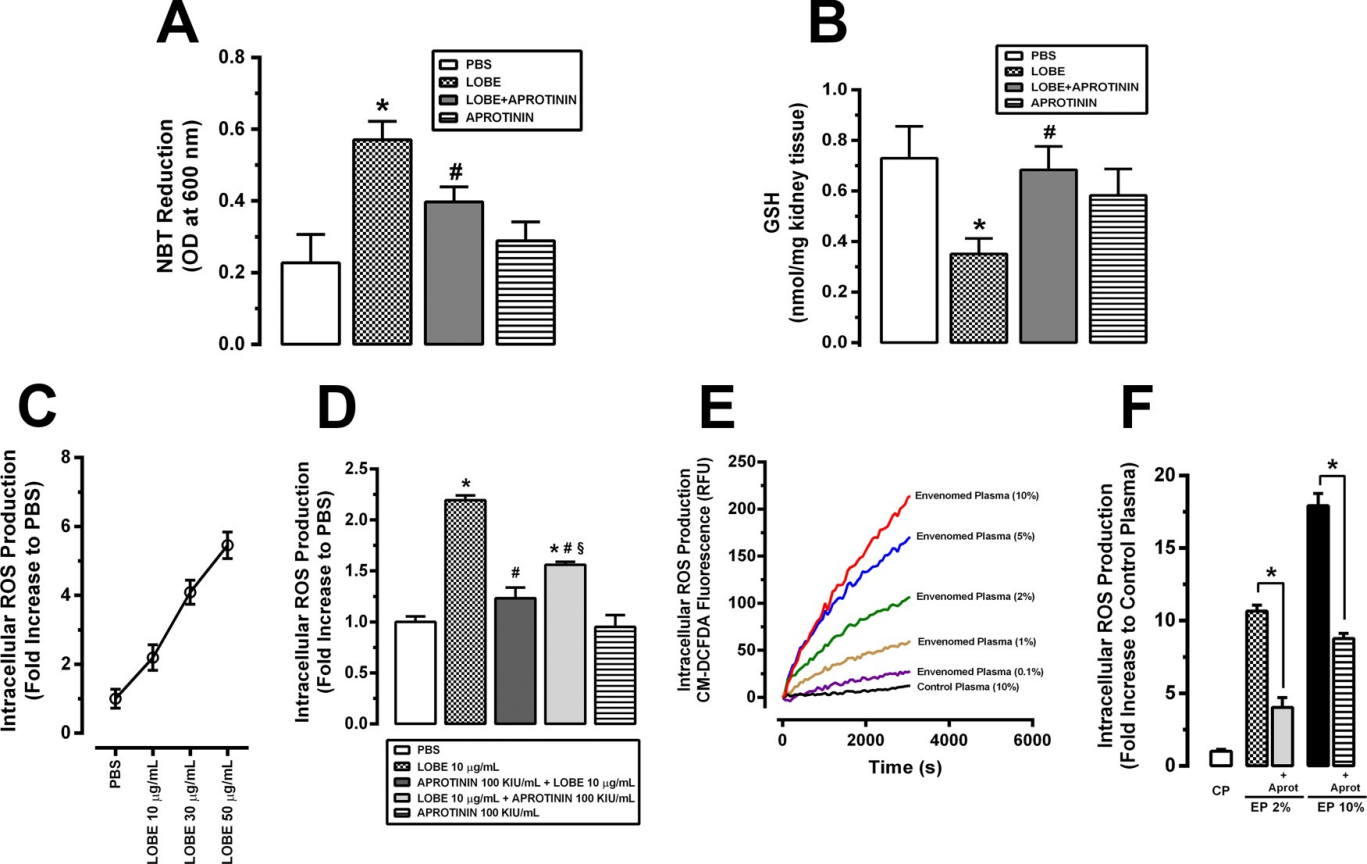
Kallikrein inhibition during *L*. *obliqua* envenomation attenuates reactive oxygen species (ROS) production *in vivo* and intracellular ROS generation on vascular smooth muscle cells (VSMC) *in vitro*. Kidneys from Wistar rats pre-treated or not with aprotinin were collected after 24h of *L*. *obliqua* venom injection and superoxide anion production **(A)** and reduced glutathione (GSH) levels **(B)** were estimated as described in material and methods. **C.** VSMC (5 x 10^3^ cells) were loaded with DCF (10 μM), stimulated with LOBE (10–50 μg/mL) and the kinetic of intracellular ROS generation was monitored by CM-H2DCFDA fluorescence. Results are expressed as fold increase to phosphate-buffered saline (PBS) stimulated cells. **D.** VSMC culture was pre-treated or not with aprotinin, loaded with DCF (10 uM) and stimulated with LOBE (10 μg/mL). Alternatively, DCF loaded VSMC were co-stimulated with LOBE+aprotinin (added at the same time) prior to CM-H2DCFDA measurement. **E.** Intracellular ROS production was monitored in DCF loaded VSMC stimulated with different concentrations (0.1–10%) of diluted plasma (1:10) from envenomed and non-envenomed rats (control animals). **F.** Intracellular ROS production was monitored in DCF loaded VSMC treated or not with aprotinin (100 KIU/mL) prior to envenomed (2 or 10%) or non-envenomed diluted plasma (control plasma) stimulation. Data represents mean ± SE. * denotes p<0.05 vs PBS group, ^#^ denotes p<0.05 vs group treated with LOBE, § denotes p<0.05 vs group treated with aprotinin 100 KIU/mL + LOBE 10 μg/mL, CP denotes control plasma, EP denotes envenomed plasma and aprot denotes aprotinin.

Regarding ROS production, it was investigated both *in vivo* in envenomed animals and *in vitro* in VSMC **(Fig 7)**. Reduced glutathione (GSH) levels, and superoxide anion levels (NBT reduction) were assessed in kidneys from envenomed rats (which received 1.5 mg/kg, s.c LOBE) pre-treated or not with aprotinin (40,000 KIU/kg, i.v) **(Fig 7A and 7B)**. LOBE induced superoxide anion production **(Fig 7A)** and reduction of GSH levels **(Fig 7B)**; both effects were prevented by aprotinin treatment. Since it is known that LOBE-induced intracellular ROS production is able to change some VSMC functions **[22]**, here we investigated whether kallikrein blockage could modulate these alterations. **Fig 7C** shows that LOBE induced a potent and dose-dependent intracellular ROS production in VSMC, as determined with a CM-H2DCFDA (DCF) probe. This effect was blocked in the presence of aprotinin, added either previously to LOBE stimulus or together with the venom in the cell cultures **(Fig 7D)**. Interestingly plasma from envenomed animals also triggered a very potent and sustained intracellular ROS production in VSMC. Even when VSMC were stimulated with low concentrations (such as 0.1 or 1%) of diluted plasma from envenomed rats (diluted 1:10), a significant intracellular ROS generation could be detected **(Fig 7E)**. Accordingly to the other results, impairing of kallikrein activation partially inhibited this effect in a dose-dependent manner **(Fig 7F)**.

### Aprotinin inhibited *L*. *obliqua* venom—Induced VSMC proliferation and migration

**Fig 8:**
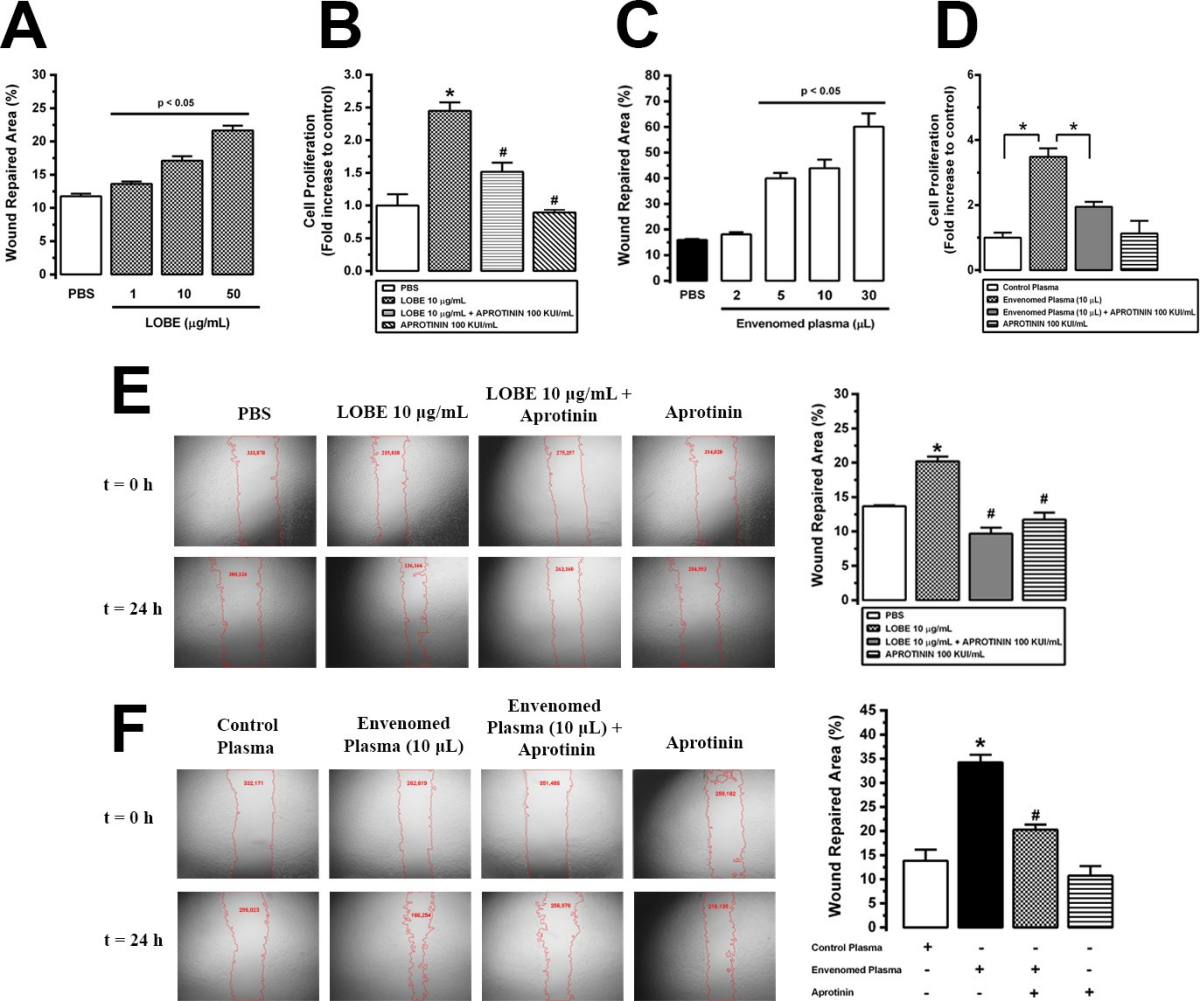
Effects of aprotinin on *L*. *obliqua* venom—induced VSMC proliferation and migration. **A.** Dose-response effect of LOBE (1–50 μg/mL) on VSMC migration was estimated by the wound healing assay. Pictures were taken at 24h after the initial scratch and the percent of wound repaired area was calculated using ImageJ software. **B.** VSMC culture was treated or not with aprotinin (100 KIU/mL) 24h prior to *L*. *obliqua* venom (10 μg/mL) addition. Cell proliferation was then estimated by MTT assay after 24h. Results are expressed as fold increase to control (cells treated with PBS). **C.** Dose-response effect of diluted plasma (1:10) from envenomed animals (1–30 μL) on VSMC migration was estimated by the wound healing assay 24h after the initial scratch. **D.** VSMC culture was treated or not with aprotinin (100 KIU/mL) 24h prior to the addition of diluted plasma (1:10) from non-envenomed (control plasma) or envenomed animals. Then, cell proliferation was determined by MTT assay after 24h. Results are expressed as fold increase to control (cells treated with control plasma). **E.** VSMC culture was treated or not with aprotinin (100 KIU/mL) 24h prior to *L*. *obliqua* venom (10 μg/mL) addition. Then, cell migration was estimated by the wound healing assay 24h after the initial scratch. **F.** VSMC culture was treated or not with aprotinin (100 KIU/mL) 24h prior to the addition of diluted plasma (1:10) from non-envenomed (control plasma) or envenomed animals. Then, cell migration was estimated by the wound healing assay 24h after the initial scratch. In E and F the panel shows representative images of wound healing assay with the respective individual values of area (numbers in red) and the calculated percent values of wound repaired area. Data represents mean ± SE. * denotes p<0.05 vs group treated with PBS or control plasma, ^#^ denotes p<0.05 vs group treated with LOBE or envenomed plasma.

Finally, we further investigated the effects of LOBE and plasma harvested from envenomed animals on VSMC migration and proliferation **(Fig 8)**. LOBE (1–50 μg/mL) directly induced VSMC migration **(Fig 8A)** and proliferation **(Fig 8B)** as estimated by the wound healing and MTT assays, respectively. Interestingly plasma from envenomed animals also stimulated VSMC migration **(Fig 8C)** and proliferation **(Fig 8D)** even when added in low doses (such as 5 μL of 1:10 diluted plasma) in comparison with cells stimulated with control healthy plasma. In another set of experiments VSMC were incubated 24 h with aprotinin before LOBE or envenomed plasma stimulation. As it can be observed in **Fig 8B and 8D** aprotinin inhibited significantly the pro-proliferative effect elicited by venom or plasma from envenomed rats. In a similar way, both LOBE or envenomed plasma stimulated VSMC migration toward wounded area after 24 h, whereas aprotinin pre-treatment decreased the wound repaired area by around of 10% in LOBE-stimulated VSMC **(Fig 8E)** and 15% in envenomed plasma-stimulated VSMC **(Fig 8F)**.

## Discussion

Collectively, our results indicate that the renal KKS is activated by *L*. *obliqua* venom in rats under experimental condition, a situation that causes development of AKI. It was also found that inhibition of the activated systemic kallikrein reduces the development of tubular injury, renal inflammation and oxidative stress thus protecting renal function against hemostatic and vascular abnormalities. Although AKI is a common consequence of accidental contact with *L*. *obliqua* caterpillars, the present study was the first to demonstrate both the importance of the KKS in venom-induced pathology and that the inhibition of kallikrein could be a promising therapeutic option to be clinically used together with antivenom immunotherapy.

To date, only animal-derived antivenoms have been provide to be effective in the treatment of different envenomation cases caused by snakes and many other venomous animals **[38]**. *L obliqua* envenomation is included in the same situation. Several laboratories throughout the continents manufacture antivenoms **[39]**, which consist of whole IgG molecules or products of their enzymatic digestion, such as bivalent F(ab')_2_ or monovalent Fab **[40–42]**. Despite its efficacy, antivenom therapy has been associated with several problems, including the high cost of production; failures in the distribution of antivenom ampoules to localized, but distant, areas that have a high incidence of accidents and/or the low-income agrarian regions in which they are most needed; inadequate storage and transportation of antivenoms; and the lack of trained health workers who know how to use these products and conduct the appropriate clinical management of medical emergencies **[39]**. Another serious problem is the adverse effects caused by antivenom therapy, including potentially life-threatening systemic disturbances, such as anaphylactic reactions, which occur quite frequently **[43]**. The beneficial effects observed following treatment with a kallikrein inhibitor in the present study are of particular interest for this reason. Microvascular thrombosis following aprotinin treatment, which could be an expected adverse effect in this type of envenomation due to fibrinolysis blockage, did not occurred in our experimental animals. Another advantage of aprotinin is that it a well-known drug, with known adverse effects and easily availability. Moreover, these results pointed out that targeting downstream components in the kallikrein cascade, such as the bradykinin receptors B_1_R and B_2_R, could also be useful, as it has been demonstrated for other pathologies such as airway inflammation, diabetic neuropathy, brain ischemia, arthritis and neuropathic pain **[12]**.

Previous data had shown that *L*. *obliqua* venom possesses high kininogenase activity, able to release BK from low molecular weight kininogen (LMWK), leading to a significant decrease in arterial blood pressure *in vivo* **[8]**. Here we demonstrate that the venom also has the ability to directly activate plasma prekallikrein *in vitro*, whose activity can be inhibited both by aprotinin or anti-lonomic serum. Kallikrein is also believed to be activated *in vivo*, because prekallikrein levels were decreased in the plasma of human victims **[7]**, renal tissue kallikrein activity and protein expression was increased in envenomed rats (as observed in the present study). In addition, the expression of rat kininogen increased in the kidneys **[6]**. Taken together, these results are in agreement with the hypothesis that BK is generated during envenomation, because *L*. *obliqua* venom can release BK directly from LMWK and indirectly through high molecular weight kininogen (HMWK), which is known to be cleaved by kallikreins. Thus, it is possible that generated BK is involved in kidney hemodynamic alterations. While BK produces a vasorelaxant effect mediated by B_2_R receptor, the BK metabolite, des-Arg^9^-BK, induces a renal vasoconstrictor response through the B_1_R receptor, leading to a decrease in glomerular filtration rate and electrolyte imbalance **[44,45]**, in a similar way to that observed in the present study.

*L*. *obliqua* envenomation is characterized by several hemostatic disturbances **[2]**. Both human patients and experimental animals displayed signs of consumption coagulopathy with blood incoagulability, prolonged coagulation times, low fibrinogen levels and platelet hypoaggregation **[25,36]**. The venom contains prothrombin and factor X activators that are able to directly activate extrinsic cascade, which ultimately contribute to fibrinogen consumption and blood incoagulability **[46,47]**. On the other hand, kallikrein is the main enzyme responsible to trigger the intrinsic pathway by directly activating factor XII **[48,49]**. Our results suggested that *L*. *obliqua* venom-induced intrinsic cascade activation is equally important for blood incoagulability, since blocking kallikrein with aprotinin protected envenomed animals against bleeding and fibrinogen consumption. An important point to be mentioned is that aprotinin can also inhibit other coagulation or fibrinolysis proteases than kallikrein, so its beneficial effects on coagulation disturbances probably are not due only by its ability to block kallikrein. However, the participation of plasma kallikrein and contact system activation is equally important, as it was demonstrated by the *in vitro* experiments of prekallikrein activation in a more purified system than rat or human plasma.

Besides its procoagulant effect in plasma, *L*. *obliqua* venom is also capable to induce expression of procoagulant and pro-inflammatory molecules such as tissue factor (TF), IL-6 and IL-1β in endothelial and VSMC, contributing to coagulation cascade activation and consequent blood incoagulability **[50,51]**. In addition, rats have more than ten members of the tissue kallikrein family and LPS-stimulated VSMC (including A7r5 cell line) showed increased expression of kininogen and the true kallikrein (encoded by *rKLK1* gene) **[52]**. In fact, in our experimental model, envenomed animals presented an increase in kidney TF activity and LOBE-stimulated VSMC *in vitro* displayed an increased kallikrein generating activity changing the cells to a procoagulant profile. It seems that kallikrein has a significant role not only in plasma coagulation, but also in modulating cell and tissue procoagulant state, since aprotinin inhibited this effect. Both cell and tissue procoagulant effect can be directly linked to glomerular function alterations observed herein. A common characteristic of venom-induced consumption coagulopathy is blood incoagulability and bleeding from large vessels, while presenting microvascular thrombosis in capillaries **[53]**. Thus, it is reasonable to propose that venom-activated VSMC contribute inducing fibrin formation and deposition in glomerular capillaries, then reducing the filtration capacities. Accordingly, previous data showed that venom toxins were immunolocalized on kidney vessels, including on VSMC, and fibrin expression increased in renal tissue from rats 24 h-post venom injection **[6]**.

An interesting finding of this work was related to the effects of plasma from envenomed animals on VSMC functions. Even diluted plasma added in small quantities to the cell was able to trigger cellular responses such as VSMC procoagulant activity, increase in ROS production, cell proliferation and migration. Among the plasma components which are expected to be involved in these cell responses are activated coagulation factors and cytokines produced during the envenomation. However, we cannot discard the participation of hemoglobin and free heme in all these events. Although in physiological conditions hemoglobin and free heme rapidly binds to serum proteins like haptoglobin and hemopexin, during extensive hemolysis the amount of cell-free hemoglobin exceeds the binding capacities of those hemoglobin-heme-scavenging mechanisms, resulting in intravascular free heme release and accumulation **[54]**. Free heme is pro-oxidant and enhances oxidative stress and inflammation. Recent studies have shown that free heme acts as a danger signal which can induce pro-inflammatory cytokine expression, ROS generation and TF-dependent coagulation activation in mice **[55,56]**. In VSMC heme induces migration and proliferation which depends on the production of NADPH oxidase (NADPHox)–derived ROS and redox-sensitive proliferation-related signaling routes, such as Mitogen Activated Protein Kinase (MAPK) and NF-kB **[57]**. In *L*. *obliqua* envenomation, the occurrence of systemic hemolysis is well documented both in envenomed patients and experimental animals **[58,59]**. Envenomed rats presented hematuria, hemoglobin deposition inside proximal and distal tubules and heme-scavenging molecules (such as hemopexin, heme-oxygenase-1 and biliverdin-reductase) was found to be up-regulated in envenomed kidneys **[6]**. Although we did not determine the levels of hemoglobin or plasma free heme in the present study, the urine from envenomed animals had a reddish-brown color and plasma hemolysis was macroscopically visual (as previously shown **[58,59]**), suggesting that heme-scavenging system is impaired. Taken together all this evidence, it’s reasonable to suppose that free heme released during the envenomation could modulate several VSMC functions, probably inducing TF, increasing kallikrein and procoagulant activity and also activating redox-sensitive proliferation and migration signaling pathways. Since kininogen and kallikrein are both expressed in VSMC **[52]** and BK promotes NADPHox activity via its GPCR receptors **[60]**, we can speculate that the mechanism involved in aprotinin effectiveness was related to the decrease in kallikrein-dependent BK and ROS generation. Besides the indirect effects on VSMC through the production of modulating molecules generated in plasma during envenomation, LOBE also acts directly on VSMC triggering cell migration, proliferation and ROS formation. Recently, it was demonstrated that the effects of LOBE on VSMC migration and proliferation is preceded by alteration in actin cytoskeleton dynamics, extracellular signal-regulated kinase (ERK) phosphorylation and ROS production via NADPHox **[22]**. Here we showed that these effects seem to be mediated by kallikrein, since its inhibition decreased migration, proliferation and intracellular-ROS formation in VSMC.

**Fig 9:**
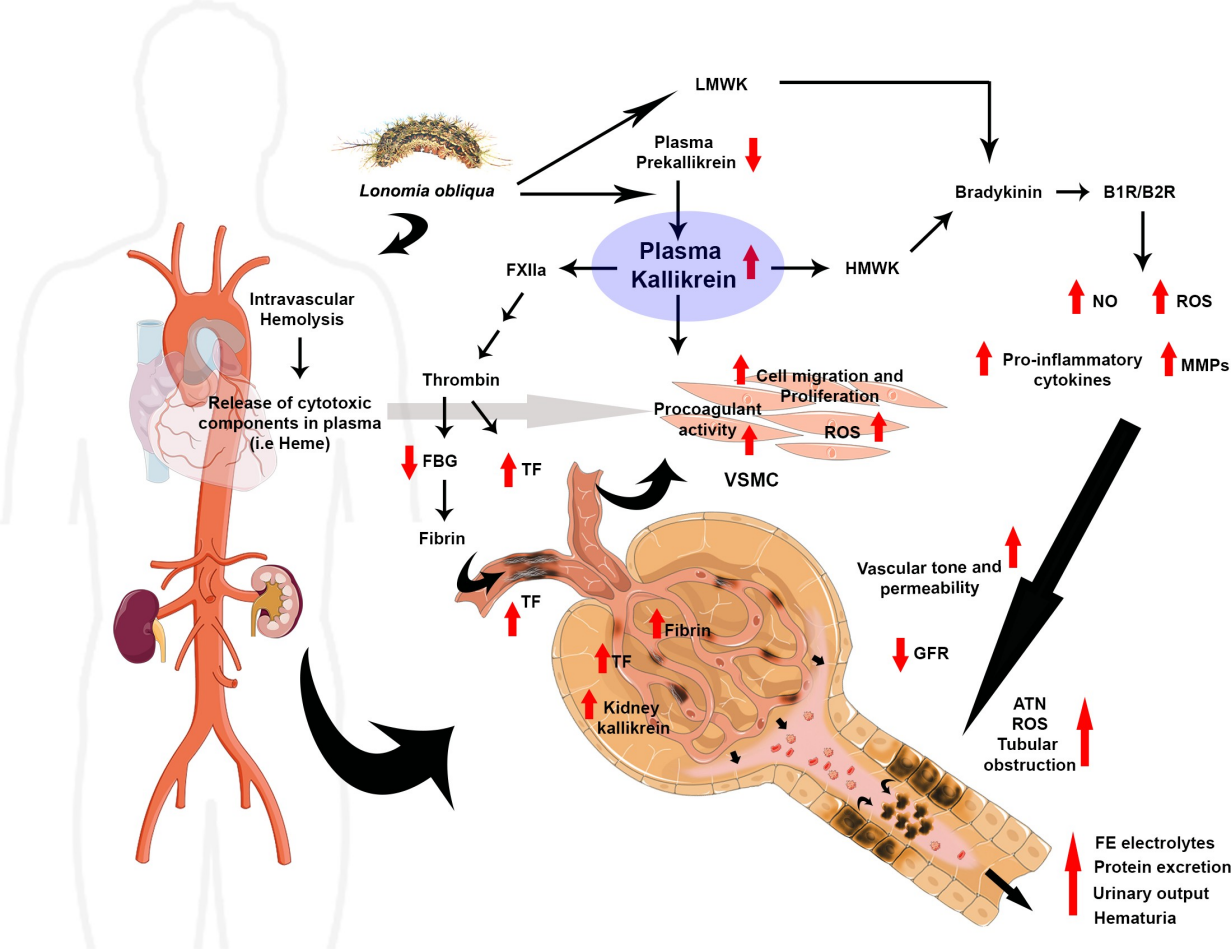
Overview about the multiple roles of kallikrein in *L*. *obliqua*–induced renal and vascular disturbances. *L*. *obliqua* venom is able to directly activate kallikrein in plasma. Consequently, prekallirein levels decrease, while increase plasma and tissue levels of active kallikrein. Once activated, it seems that kallikrein participate in kidney and vascular disturbances by two main mechanisms: (*i*) Triggering the intrinsic pathway of coagulation and activating VSMC changing it to a procoagulant profile. Both mechanisms lead to the formation of procoagulant enzymes and up-regulation of TF, which ultimately are involved in fibrinogen consumption and deposition of fibrin in glomerular small vessels; and (*ii*) Releasing bradykinin (BK) from LMWK and HMWK. BK acts through activation of the B1 and B2 receptors being involved in inflammation, ROS and NO production, vascular remodelling, control of vascular tone and permeability and also in electrolyte imbalance. Altogether, probably these vascular alterations contribute to a significant decrease in renal blood flow and perfusion pressure which is accompanied by ATN, tubular obstruction (caused by cell debris accumulated inside tubules) and a reduction in GFR. Evidence also points to the participation of cytotoxic components released in plasma during envenomation due to intravascular hemolysis. These components participate increasing cell migration, proliferation and activating kallikrein-mediated ROS sensitive signalling pathways in VSMC. Red arrows denote increase or decrease of components and process determined in the present study. Abbreviations: FBG, fibrinogen; ATN, acute tubular necrosis; TF, tissue factor; LMWK, low molecular weight kininogen; HMWK, high molecular weight kininogen; ROS, reactive oxygen species; NO, nitric oxide; MMPs, matrix metalloproteinases; VSMC, vascular smooth muscle cells; GFR, glomerular filtration rate.

In some pathological conditions such as in renal ischemia-reperfusion, glomerulonephritis and obstructive nephropathy, once released by kallikrein, BK contributes to kidney injury mainly by activation of its two receptors, B1R and B2R **[61–63]**. Signaling through BK receptors leads to mobilization of nuclear factor - κB (NF-κB), expression of TNF-α and IL-1β, NO, ROS and matrix metalloproteinases (MMPs), which will ultimately participate in tissue remodeling, vascular leakage, hemodynamic and kidney electrolyte dysfunction **[13]**. **Fig 9** summarizes these cellular and molecular connections. In envenomed animals up-regulation of several proteins associated with the acute phase of inflammatory signaling and nephritis have been described in the kidney **[6]**. *In vitro*, when incubated with human fibroblasts or endothelial cells in culture, *L*. *obliqua* venom induces the production of several cytokines, including TNF-α and IL-1β, and also activates nuclear factor - κB (NF-κB) and increases the expression of inflammatory inducible enzymes, such as cyclooxygenase– 2 (COX-2), inducible nitric oxide synthase (iNOS), heme-oxygenase (HO-1) and MMPs **[50,64]**. Regarding tissue oxidative stress *in vivo*, kidney of envenomed animals presented increased levels of superoxide, NO, MMPs and reduced levels of reduced glutathione (GSH). All these alterations were also accompanied by tubular lesions, accumulation of pro-inflammatory cells and release of cytokines such as TNF-α, which is a known inductor of kininogen and bradykinin B1R receptor expression **[11]**. Collectively all the evidence points to an activation of kininogen-kallikrein-BK-B1R/B2R axis during *L*. *obliqua* envenomation, since blocking the initial step of this pathway with aprotinin improves renal and vascular function **(Fig 9)**.

An overview about the multiple effects of kallikrein on *L*. *obliqua* venom-induced kidney and vascular injury is presented in **Fig 9**. Taken together, the results revealed that kallikrein inhibition improves renal and vascular function during *L*. *obliqua*-induced AKI by reducing tubular necrosis, renal inflammation, and the production of pro-inflammatory cytokines, and ameliorates the coagulopathy commonly observed during this type of envenomation. Venom-induced kallikrein also modulates VSMC functions such as migration, proliferation and ROS-sensitive signaling pathways. Thus, the present study provides consistent evidence linking the kallikrein-kinin system activation to *L*. *obliqua*-induced AKI and indicates that the inhibition of its components may constitute an efficient therapeutic alternative that can be used to control the progression of renal injury during such envenomation.

## Supporting information

S1 Methods Detailed description of prekallikrein production and prothrombin/factor X deficient plasma assays. (DOCX)

S1 Fig Prekallikrein identification and purification. Plasma prekallikrein (PPKLK) was purified from rat plasma through steps of ammonium sulfate precipitation followed by chromatographic steps on DEAE, heparin and CM-sepharose. The homogeneity of preparation (25 μg) was analyzed by SDS-PAGE 12% under reducing conditions and the identity of PPKLK was confirmed by western-blot using a specific anti–rat PPKLK antibody.
(TIF)

S2 Fig LOBE can not release procoagulant factors from prekallikrein (PKLK) preparation. In order to confirm that our PKLK preparation was free from procoagulant zymogens, different LOBE concentrations were incubated in the presence or absence of purified PKLK and the release of procoagulant enzymes were tested by adding *p*-nitroanilide based chromogenic substrates designed by FXa **(A)** or thrombin **(B)**. In both cases, the kinetics of *p*-nitroaniline formation were monitored at 405 nm and results expressed as mOD/min. Data on curves represents mean of three independent experiments ± SE.
(TIF)

S3 Fig LOBE-induced kallikrein generation in human normal plasma. Human plasma was diluted (1:10) in PBS and incubated with different concentrations of *L*. *obliqua* bristle extract (LOBE) in the presence of 100 nM SBTI in a final volume of 100 μL, at 37 ^o^C. Aliquots of 10 μL were taken and generated kallikrein enzymatic activity was determined using the specific chromogenic substrate S2302 (2 mM). The kinetics of p-nitroaniline formation were monitored at 405 nm and curves are representative data from at least three independent experiments. Inset shows the dose-response curve. The amounts of plasma derived kallikrein generated by LOBE was estimated using a calibration curve made with known concentrations of purified kallikrein and thus expressed as pmol of equivalent kallikrein/mL/min.
(TIF)

S4 Fig LOBE-induced kallikrein generation in factor X and prothrombin deficient plasma. To further confirm LOBE-induced kallikrein activation specificity, the main procoagulant factors, FX and prothrombin (PThr), were depleted from human plasma, generating a FX and PThr deficient plasma (-FX/-PThr). **A.** Deficient plasma (-FX/-PThr) was diluted (1:10) in PBS, activated with ellagic acid in the presence of calcium ions and kallikrein, FXa and thrombin-like generated activities were measured by adding the specific chromogenic substrates. **B.** Diluted deficient plasma (-FX/-PThr) was incubated in the presence and absence of LOBE (50 μg/mL) or aprotinin (100 KIU/mL) and kallikrein-like activity was then measured by the addition of S-2302 substrate. **C.** Diluted deficient plasma (-FX/-PThr) was incubated in the presence and absence of LOBE (50 μg/mL) and factor Xa-like activity was then measured by the addition of S-2222 substrate. **D.** Diluted deficient plasma (-FX/-PThr) was incubated in the presence and absence of LOBE (50 μg/mL) and thrombin-like activity was then measured by the addition of S-2238 substrate. **E.** Diluted normal or deficient plasma (-FX/-PThr) were incubated in the presence or absence of LOBE (50 μg/mL) and generated thrombin was specifically measured through fibrin formation after addition of fibrinogen (200 μg/mL). In all cases, the curves are representative data from at least three independent experiments.
(TIF)
