# Supplementary material for: Renal and vascular effects of kallikrein inhibition in a model of Lonomia obliqua venom-induced acute kidney injury
Source: PLoS Negl Trop Dis. 2019 Feb 14;13(2):e0007197. doi: 10.1371/journal.pntd.0007197 (PMC6392336; doi:10.1371/journal.pntd.0007197)
Supplement: S1 Methods — (DOCX) [file pntd.0007197.s001.docx]

**S1 Methods**

*Prekallikrein purification*

Prekallikrein (PPKLK) was purified following the classical protocol by Heimark and Davie, 1981 with some modifications. Briefly, 50 mL of heparinized rat plasma was mixed at 4^0^C with 5 mM benzamidine, 5 mM EDTA, 1 mM EGTA, 100 mg/L soybean trypsin inhibitor (SBTI), 100 mg/L polybrene and incubated with 1/15 (v/v) 1 M BaCl_2_. The precipitate was removed by centrifugation and to the supernatant was added solid ammonium sulfate slowly until reach 20 % of saturation. The mixture was then stirred for 30 min at 4^0^C, the precipitate was removed and the supernatant was brought to 40 % saturation with solid ammonium sulfate. In this step, the precipitate was recovered, dissolved (10 mL) in 50 mM Tris-HCl, 5 mM benzamidine, 5 mM EDTA, 1 mM EGTA, 50 mg/L polybrene and 20 mg/L SBTI, pH 7.5 and dialyzed against the same buffer containing additionally 50 mM NaCl to completely remove ammonium sulfate. The sample was then applied to the first chromatographic step on DEAE-sepharose equilibrated in 50 mM Tris-HCl, 50 mM NaCl, pH 7.5. Under these conditions, PPKLK passes directly through the DEAE-sepharose column, while factor XII remains adsorbed. The fraction enriched in PPKLK was applied to a second chromatographic step on heparin-sepharose equilibrated in 50 mM Tris-HCl, 50 mM NaCl, pH 7.5 and the fractions were eluted by a linear salt gradient from 50 mM to 500 mM NaCl in the same buffer. This step eliminates most of the factor XI contaminants. Lastly, high molecular weight kininogen contaminants were eliminated by a third chromatographic step on CM-sepharose equilibrated in 50 mM sodium acetate, pH 5.6. PPKLK was eluted by a linear gradient from 0 to 100 mM NaCl in the same buffer. PPKLK containing fractions were pooled, concentrated and its homogeneity and identity was verified by SDS-PAGE and immunoblot using a rabbit anti-rat polyclonal PPKLK antibody as described in material and methods section. All chromatographic steps were performed on a FPLC system (Amersham Biosciences, Uppsala, Sweden) and run in the presence of polybrene to avoid contact activation.

*Prothrombin and factor X deficient plasma*

A human plasma depleted in prothrombin and factor X (plasma -PThr/-FX) was prepared from a commercially available factor X deficient plasma (Sigma-Aldrich, Saint Louis, MO, USA), according to Liska and Suttie, 1988. For this purpose, factor X deficient plasma (5 mL) was mixed with solid barium sulfate (0.5 g) + magnesium sulfate (12 mg) for 1 h at 4 ^0^C under gentle agitation. Then, the supernatant was recovered by centrifugation and this procedure with barium sulfate addition was repeated three times more for prothrombin adsorption. To confirm PThr/FX depletion, plasma -PThr/-FX was diluted 1:10 in PBS and activated with a mixture containing 100 µM ellagic acid, phospholipids, 0.005 % bovine albumin and 10 mM CaCl_2_ in 50 mM Tris-HCl, pH 7.4 for 10 min at 37 °C. Kallikrein, factor Xa and thrombin – like activities were detected by the addition of 0.2 mM S2302, S2222 or S2238 chromogenic substrates, respectively. The kinetics of *p*-nitroaniline release were monitored at 405 nm for 30 min using a microplate reader spectrophotometer (SpectraMAX, Molecular Devices Co., Sunnyvale, USA).

*L. obliqua venom – induced prekallikrein activation on prothrombin and factor X deficient plasma*

Plasma -FX/-PThr was diluted as described above and 10 μL was incubated in the presence and absence of LOBE (50 μg/mL) or aprotinin (100 KIU/mL) in a final volume of 100 μL and kallikrein, factor Xa or thrombin-like activities were measured by the addition of 0.2 mM S2302, S2222 or S2238 chromogenic substrates, respectively. The kinetics of *p*-nitroaniline release were monitored at 405 nm for 30 min using a microplate reader spectrophotometer (SpectraMAX, Molecular Devices Co., Sunnyvale, USA). The intrinsic activity of LOBE (50 μg/mL) on synthetic substrates was also registered. In another set of experiments, 10 μL of diluted normal or deficient plasma (-FX/-PThr) were incubated in the presence or absence of LOBE (50 μg/mL) and generated thrombin was specifically measured through fibrin formation after addition of fibrinogen (200 μg/mL). In all assays, data are presented as representative curves of at least three independent experiments.

**References cited in S1 methods**

Heimark RL, Davie EW. Bovine and human plasma prekallikrein. Methods Enzymol. 1981; 80: Pt C:157-72.

Liska DJ, Suttie JW. Location of gamma-carboxyglutamyl residues in partially carboxylated prothrombin preparations. Biochemistry 1988; 27: 8636-41.
