# Supplementary figures and images for: Renal and vascular effects of kallikrein inhibition in a model of Lonomia obliqua venom-induced acute kidney injury
Source: PLoS Negl Trop Dis. 2019 Feb 14;13(2):e0007197. doi: 10.1371/journal.pntd.0007197 (PMC6392336; doi:10.1371/journal.pntd.0007197)

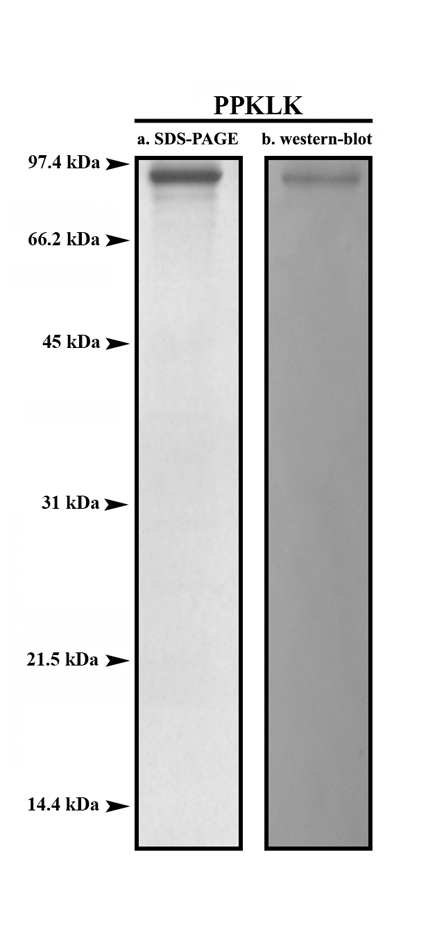

Supplement: S1 Fig — Plasma prekallikrein (PPKLK) was purified from rat plasma through steps of ammonium sulfate precipitation followed by chromatographic steps on DEAE, heparin and CM-sepharose. The homogeneity of preparation (25 μg) was analyzed by SDS-PAGE 12% under reducing conditions and the identity of PPKLK was confirmed by western-blot using a specific anti–rat PPKLK antibody. (TIF) [file pntd.0007197.s002.tif]

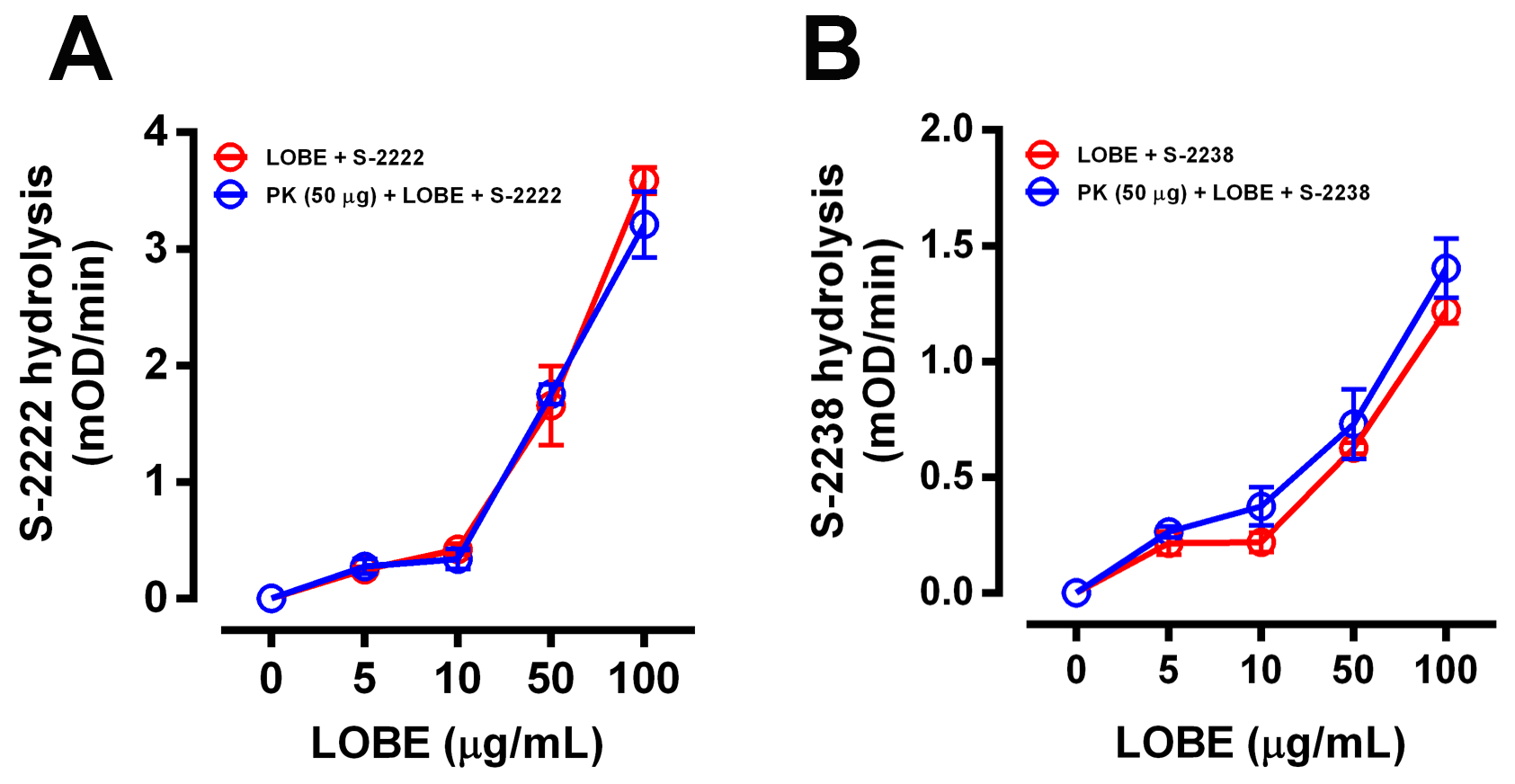

Supplement: S2 Fig — In order to confirm that our PKLK preparation was free from procoagulant zymogens, different LOBE concentrations were incubated in the presence or absence of purified PKLK and the release of procoagulant enzymes were tested by adding p-nitroanilide based chromogenic substrates designed by FXa (A) or thrombin (B). In both cases, the kinetics of p-nitroaniline formation were monitored at 405 nm and results expressed as mOD/min. Data on curves represents mean of three independent experiments ± SE. (TIF) [file pntd.0007197.s003.tif]

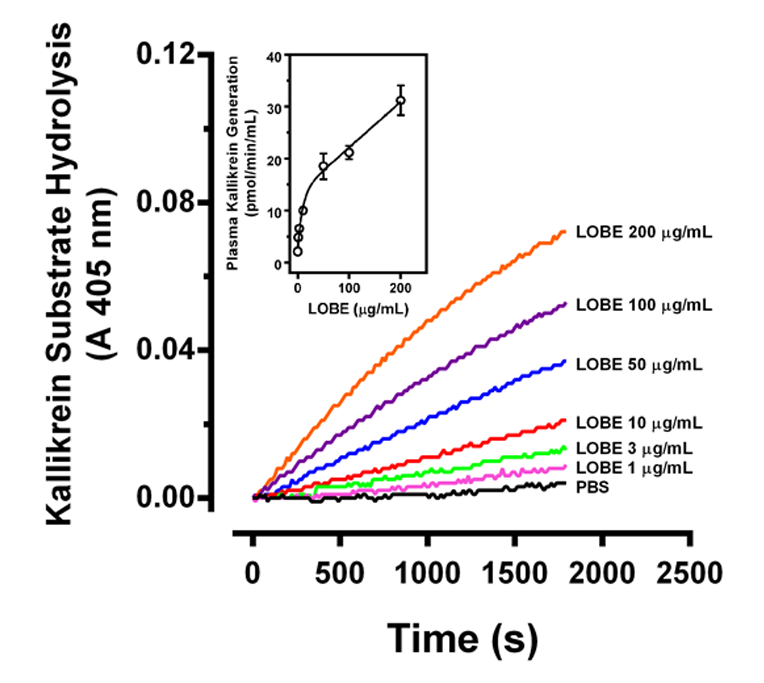

Supplement: S3 Fig — Human plasma was diluted (1:10) in PBS and incubated with different concentrations of L. obliqua bristle extract (LOBE) in the presence of 100 nM SBTI in a final volume of 100 μL, at 37 oC. Aliquots of 10 μL were taken and generated kallikrein enzymatic activity was determined using the specific chromogenic substrate S2302 (2 mM). The kinetics of p-nitroaniline formation were monitored at 405 nm and curves are representative data from at least three independent experiments. Inset shows the dose-response curve. The amounts of plasma derived kallikrein generated by LOBE was estimated using a calibration curve made with known concentrations of purified kallikrein and thus expressed as pmol of equivalent kallikrein/mL/min. (TIF) [file pntd.0007197.s004.tif]

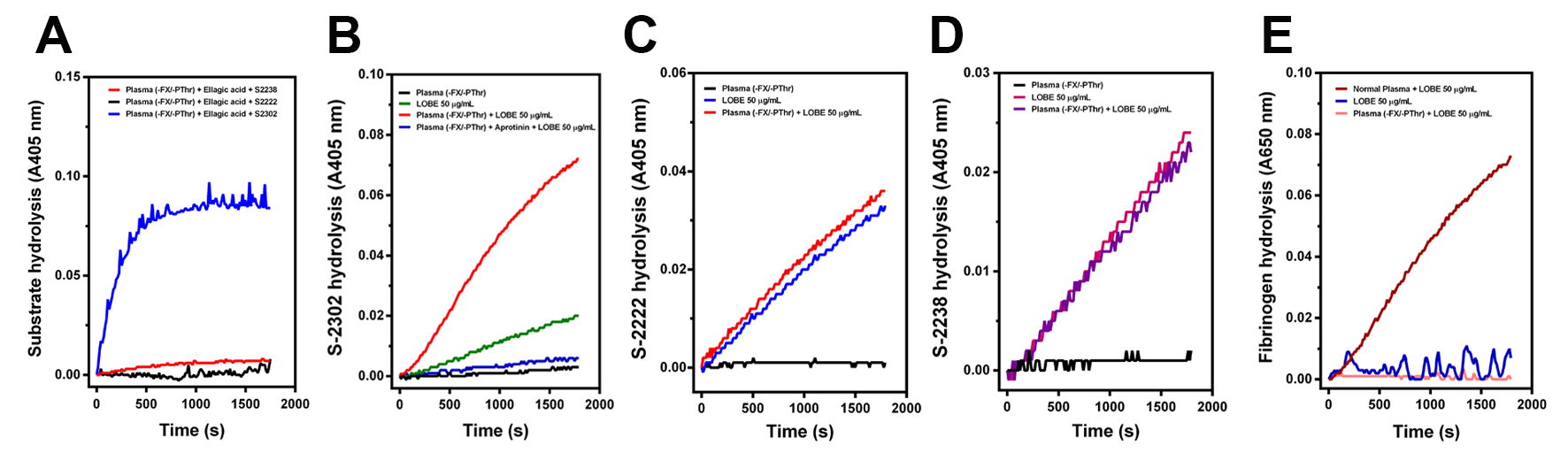

Supplement: S4 Fig — To further confirm LOBE-induced kallikrein activation specificity, the main procoagulant factors, FX and prothrombin (PThr), were depleted from human plasma, generating a FX and PThr deficient plasma (-FX/-PThr). A. Deficient plasma (-FX/-PThr) was diluted (1:10) in PBS, activated with ellagic acid in the presence of calcium ions and kallikrein, FXa and thrombin-like generated activities were measured by adding the specific chromogenic substrates. B. Diluted deficient plasma (-FX/-PThr) was incubated in the presence and absence of LOBE (50 μg/mL) or aprotinin (100 KIU/mL) and kallikrein-like activity was then measured by the addition of S-2302 substrate. C. Diluted deficient plasma (-FX/-PThr) was incubated in the presence and absence of LOBE (50 μg/mL) and factor Xa-like activity was then measured by the addition of S-2222 substrate. D. Diluted deficient plasma (-FX/-PThr) was incubated in the presence and absence of LOBE (50 μg/mL) and thrombin-like activity was then measured by the addition of S-2238 substrate. E. Diluted normal or deficient plasma (-FX/-PThr) were incubated in the presence or absence of LOBE (50 μg/mL) and generated thrombin was specifically measured through fibrin formation after addition of fibrinogen (200 μg/mL). In all cases, the curves are representative data from at least three independent experiments. (TIF) [file pntd.0007197.s005.tif]
